# Preparation of Enzyme-Activated Thapsigargin Prodrugs by Solid-Phase Synthesis

**DOI:** 10.3390/molecules23061463

**Published:** 2018-06-15

**Authors:** Tomas Zimmermann, Søren Brøgger Christensen, Henrik Franzyk

**Affiliations:** Department of Drug Design and Pharmacology, Faculty of Health and Medical Sciences, University of Copenhagen, Jagtvej 162, DK-2100 Copenhagen, Denmark; tomas.zimmermann@vscht.cz (T.Z.); soren.christensen@sund.ku.dk (S.B.C.)

**Keywords:** prodrug, targeted chemotherapy, thapsigargin, mipsagargin, solid-phase peptide synthesis: cytotoxin-peptide conjugation

## Abstract

Since cells in solid tumors divide less rapidly than cells in the bone marrow or cells of the immune system, mitotic inhibitors often cause severe side effects when used for treatment of diseases like prostate cancer and breast cancer. One approach to overcome this problem involves attempts at developing drugs based on general cytotoxins, like calicheamicin and thapsigargin, which kill cells at all phases of the cell cycle. However, such toxins can only be used when efficient targeting to the malignant tissue is possible. In the case of thapsigargin, selectivity for tumor-associated cells is achieved by conjugating the drug to a peptide that is only cleaved in the vicinity of tumors to release the cytotoxic drug or an analog with retained activity. Solid-phase synthesis protocols were developed for preparation of three already validated prodrugs of thapsigargin: one prodrug cleavable by human kallikrein 2, one prodrug cleavable by prostate-specific antigen, and one prodrug cleavable by prostate-specific membrane antigen.

## 1. Introduction

A major problem in the treatment of cancer diseases is targeting the chemotherapeutics toward malignant cells without affecting benign cells. For a number of chemotherapeutics, like mitotic inhibitors [1,2,3], topoisomerase inhibitors [4,5], and antimetabolites [6], therapeutic efficacy relies on interference with the rapid proliferation of cancer cells during the mitotic phases of their cell cycle.

Common side effects associated with mitotic inhibitors comprise concomitant damage to proliferative cells like the bone marrow or cells of the immune system [2,7]. Moreover, such drugs are inconvenient for the treatment of solid tumors, in which many of the cells divide slowly [8]. Solid tumors develop in a number of cancer diseases, like lung cancer and prostate cancer. In these cases, cytotoxins that kill the cells both in the proliferative and quiescent phases may be preferred [8].

Natural products constitute an important source for chemotherapeutics—in particular, against cancer and diseases caused by parasites, such as malaria, onchoceriasis, and elephantitis [9]. Prominent examples among such chemotherapeutics comprise the camptothecins and the taxols (e.g., paclitaxel) [10,11] as well as vinblastine and vincristine [2]. However, in general, genuine natural products have turned out to be poor drugs, either because they have a narrow therapeutic window or because they possess undesired pharmacokinetic properties, such as poor absorption, low solubility, and/or fast metabolism. An example of a chemotherapeutic developed by the optimization of a natural product is etoposide [1].

**Figure 1:**
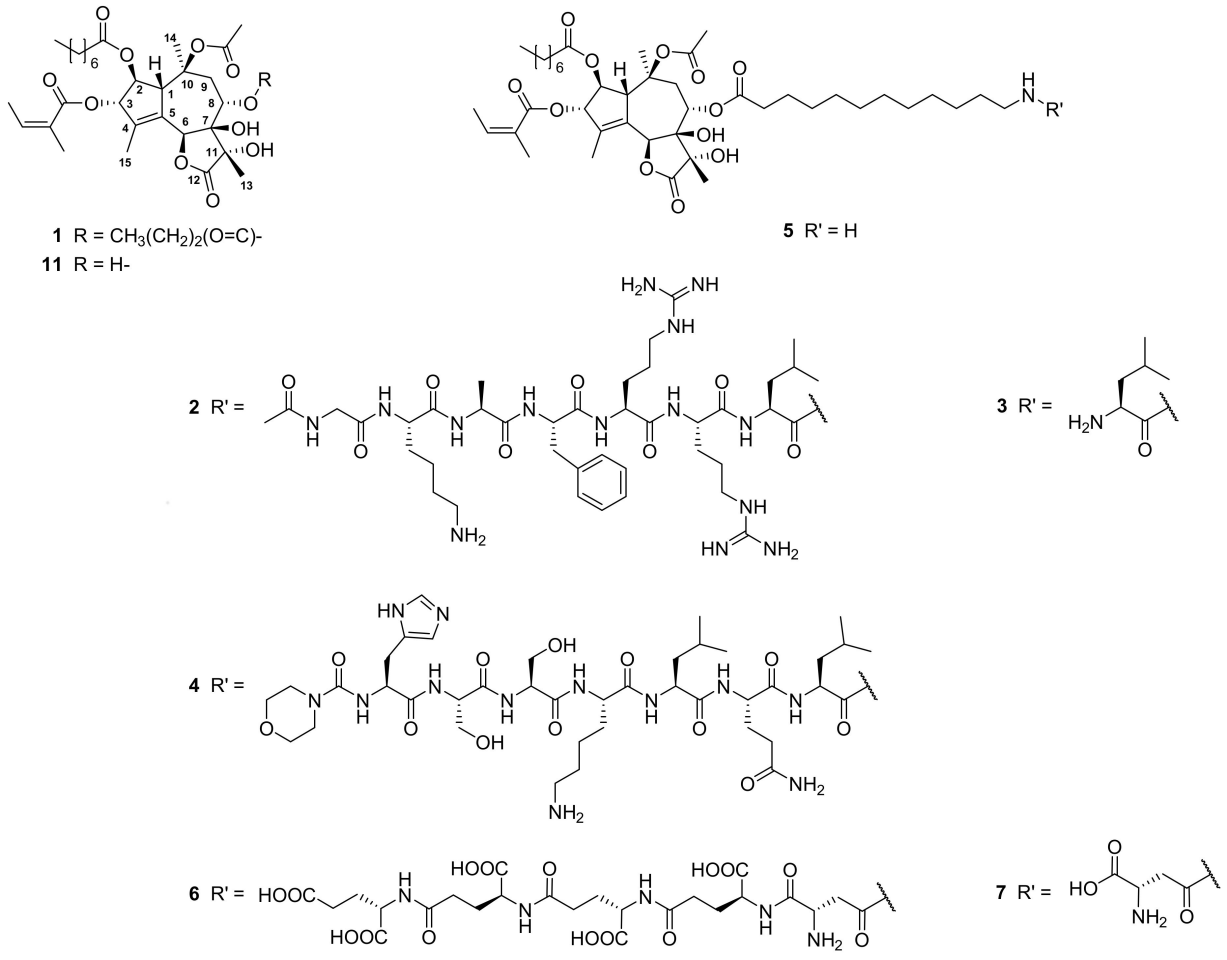
Structures of thapsigargin (**1**), 8-*O*-(12-aminododecanoyl)-8-*O*-debutanoylthapsigargin (**5**) and the prodrugs G114 (**2**), G115 (**4**), G202 (Mipsagargin, **6**), and the cleavage products **3** and **7** as well as 8-*O*-debutanoylthapsigargin (**11**).

Thapsigargin (**1**) is a potent cytotoxin isolated from *Thapsia garganica* (L.) (Figure 1) [12], and this compound induces apoptosis in a proliferation-independent way by emptying the endoplasmic reticulum of Ca^2+^ ions [13]. Thapsigargin (**1**) kills all cells, in contrast to paclitaxel, doxorubicin, and the vinca alkaloids, which preferentially kill cells during proliferation, and consequently, it cannot be used directly for systemic application.

A prerequisite for the chemotherapeutic use of non-selective cytotoxins is that targeting against the malignant tissue is possible. The prodrug approach enables targeting of toxins by taking advantage of the unique properties of cancer cells. Ideally, a prodrug should, by itself, exhibit no or very limited biological activity, whereas it is essential that selective cleavage or uptake occurs in the malignant tissue, thereby releasing an inactive promoiety and the active drug. Conjugation of cytotoxic compounds to antibodies displaying selective binding to proteins present on the surface of cancer cells has, in some cases, led to promising drugs [7,8,14,15]. Another option involves conjugation to an enzyme possessing unique substrate specificity to an antibody targeting cancerous tissue. Administration of a prodrug cleaved by the antibody-conjugated enzyme, but not by any endogenous enzyme, will only be cleaved in the malignant tissue, and consequently the toxic effect is targeted [7,8].

An alternative strategy takes advantage of proteolytic enzymes that are preferentially expressed on the surfaces of cancer cells or secreted by cells in solid tumors. If these enzymes cleave substrates that are not cleaved by other enzymes in the host, the conjugation of drugs to such substrates will afford prodrugs only cleaved in the tumor. The three enzymes—human glandular kallikrein (hK2), prostate-specific antigen (PSA), and prostate-specific membrane antigen (PSMA)—all fulfill this requirement [16,17,18,19]. PSA and hK2 are closely related proteolytic enzymes which are both excreted in the prostate. Both of these enzymes are also expressed by most prostate cancer cells, including metastatic tumors [19]. Trace amounts of PSA have also been found in other tissues, like the ileum and the epididymis, where its physiological function is to regulate the viscosity of semen [18].

The trypsin-like proteolytic enzyme hK2 cleaves the amides within Arg-Arg or Arg-Lys motifs [20]. In an elaborate study, peptide substrates for hK2 with retained stability toward other trypsin-like proteases were identified through the screening of a peptide library with the amino acid sequence GKAXXX (where X = any proteinogenic amino acid except cysteine). This revealed that Ac-GKAFRRL possessed optimal stability toward serum proteases while being labile to cleavage by hK2. Consequently, cytotoxic natural product **1** was conjugated to a peptide identified by this procedure to give prodrug **2** [20]. Cleavage of **2,** catalyzed by hK2, has been shown to give **3** with a cytotoxicity similar to that of **1** [20].

PSA is a proteolytic enzyme with chymotrypsin-like activity, and its preferred cleavage sites are between Glu and Ser or Leu [7]. Again, the screening of a number of peptides led to the design of prodrug **4**, a substrate for PSA [16] that upon cleavage also gives drug **3**. Based on an analysis of the preferred amino acids in the various positions of PSA substrates that retain PSA-specific cleavage, prodrug **4** was designed [7,16].

Angiogenetic tissue in a number of tumors, like breast cancer, prostate cancer, kidney cancer, liver cancer, and other tumors, overexpresses prostate-specific membrane antigen (PSMA) [7,16]. PSMA is a carboxypeptidase that preferentially cleaves terminal oligoglutamate γ-linked residues [21]. In healthy individuals, PSMA is mainly expressed in the prostate, but detectable levels are found in the brain and brush border cells of the small intestine [7,21]. Its poor endogenous expression outside the prostate in healthy individuals makes this enzyme interesting for the targeting of prodrugs to solid tumors. The absence of amino groups in **1** prevents its immediate conjugation to peptides; however, replacing the 8-*O*-butanoyl group with a 12-aminododecanoyl moiety to give **5** enables conjugation to a suitable peptide acid. At the same time, this spacer introduces a distance between the peptide and the bulky guaianolide skeleton, ensuring that the resulting prodrug may be a substrate for an enzyme. The conjugation of **5** with the peptide (γGlu)_4_-β-Asp gives prodrug **6** (Mipsagargin, G202) which was cleaved by PSMA to give compound **7** [16]. Mipsagargin has, in clinical phase 1, demonstrated an acceptable tolerability and a favorable pharmacokinetic profile for the treatment of refractory, advanced, or metastatic tumors [22].

Previously, solution-phase chemistry has been used for the preparation of prodrugs **2** [23], **4** [19], and **6** [16]. In the present work, we report on protocols that allow convenient preparation of these prodrugs, either by continuous synthesis or in combination with a final solution-phase linkage step. These methods are considered to be readily adapted to the preparation of other thapsigargin-peptide conjugates that display different linkers and/or targeting peptides.

## 2. Results and Discussion

Combined Solid-Phase and Solution-Phase Protocols for the Synthesis of Thapsigargin Conjugates and Prodrugs

The synthesis of amino acid conjugates with 12-aminododecanoyl derivative **5** to give compounds **3** and **7** has previously been reported; however, **7** displayed poorer cytotoxic activity than **3** [24]. The zwitterionic structure of **7** at physiologic pH might prevent its diffusion into cells, consequently resulting in reduced cytotoxicity. Acetylation of the amino group in the Asp residue of **7** (to give **8**) eliminates the zwitterionic structure.

**Scheme 1:**
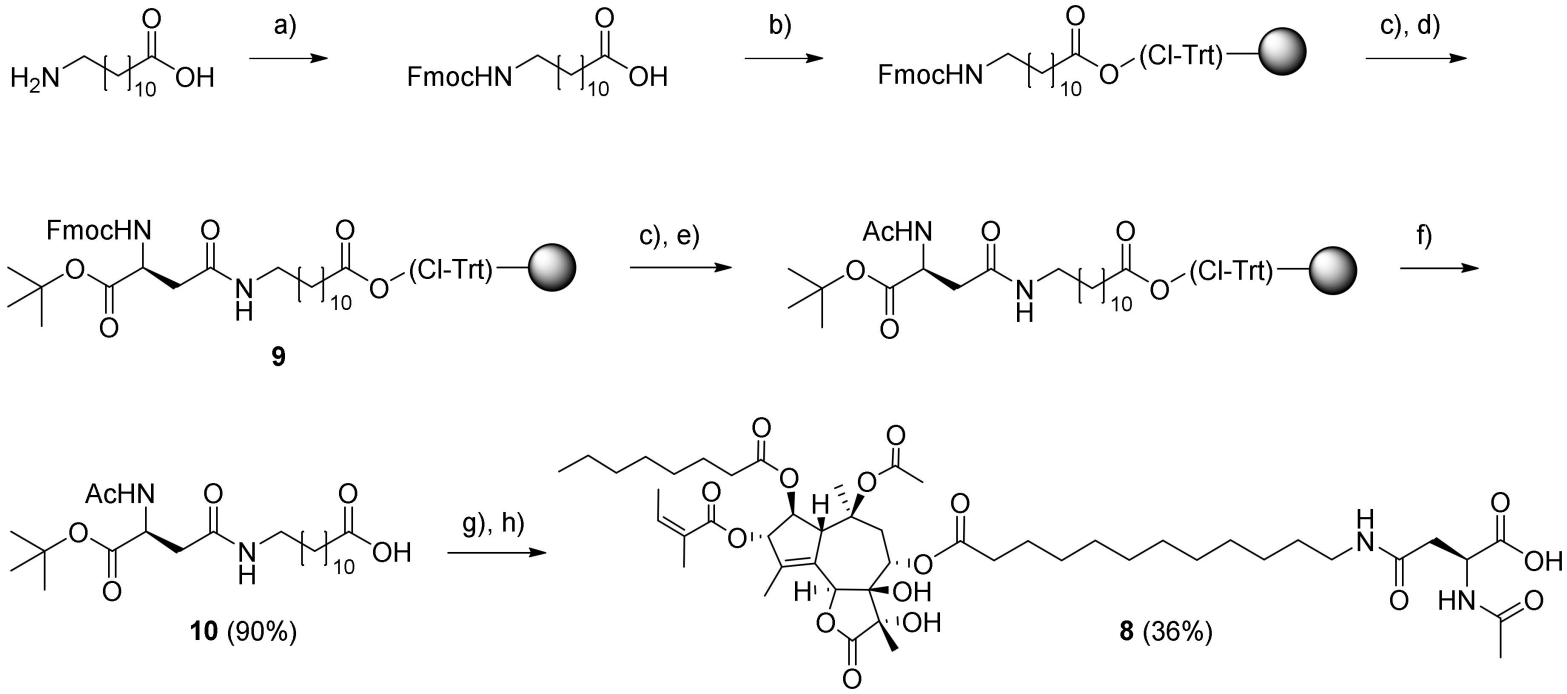
Synthesis of amino acid conjugate **8**. Reagents and conditions: (**a**) Fmoc-OSu, NaHCO_3_, H_2_O–Acetone (1:1), 20 h; (**b**) 2-chlorotrityl chloride resin, DIPEA, dichloromethane (DCM), 3 h; (**c**) 20% piperidine–DMF, 2 × 20 min; (**d**) Fmoc-Asp-O*t*Bu, PyBOP, DIPEA, DMF, 16 h; (**e**) Ac_2_O–DIPEA–NMP (1:2:3, 2 × 5 min; (**f**) 20% hexafluoroisopropanol (HFIP)–DCM, 3 × 30 min; (**g**) 1-ethyl-3-(3-dimethylaminopropyl) carbodiimide (EDC), DMAP, **8** DCM, 14 h; (**h**) trifluoroacetic acid (TFA)–DCM (2:1), 1 h; all reactions were carried out at room temperature (rt).

The preparation of resin-bound intermediate **9** was performed with N^α^-Fmoc-protected Asp with a *tert*-butyl ester on the α-carboxylic group and N-Fmoc-protected 12-aminododecanoic acid as starting materials (Scheme 1). The latter was loaded onto 2-chlorotrityl chloride (CTC) resin, and following Fmoc removal, coupling with Fmoc-Asp-O*t*Bu (using PyBOP activation) was performed. Acetylation of **9** was accomplished by successive N^α^-Fmoc deprotection and treatment with Ac_2_O-diisopropylethylamine (DIPEA) in N-methyl-2-pyrrolidone (NMP). Then, a final selective cleavage of the product from the resin with dilute hexafluoroisopropanol (HFIP) gave **10**. Esterification of *O*-8-debutanoyl-thapsigargin (**11**) with **10** using 1-ethyl-3-(3-dimethylaminopropyl) carbodiimide (EDC) in the presence of 4-(dimethylamino)pyridine in dichloromethane (DCM) and subsequent deprotection of the *tert*-butyl ester gave compound **8**.

**Scheme 2:**
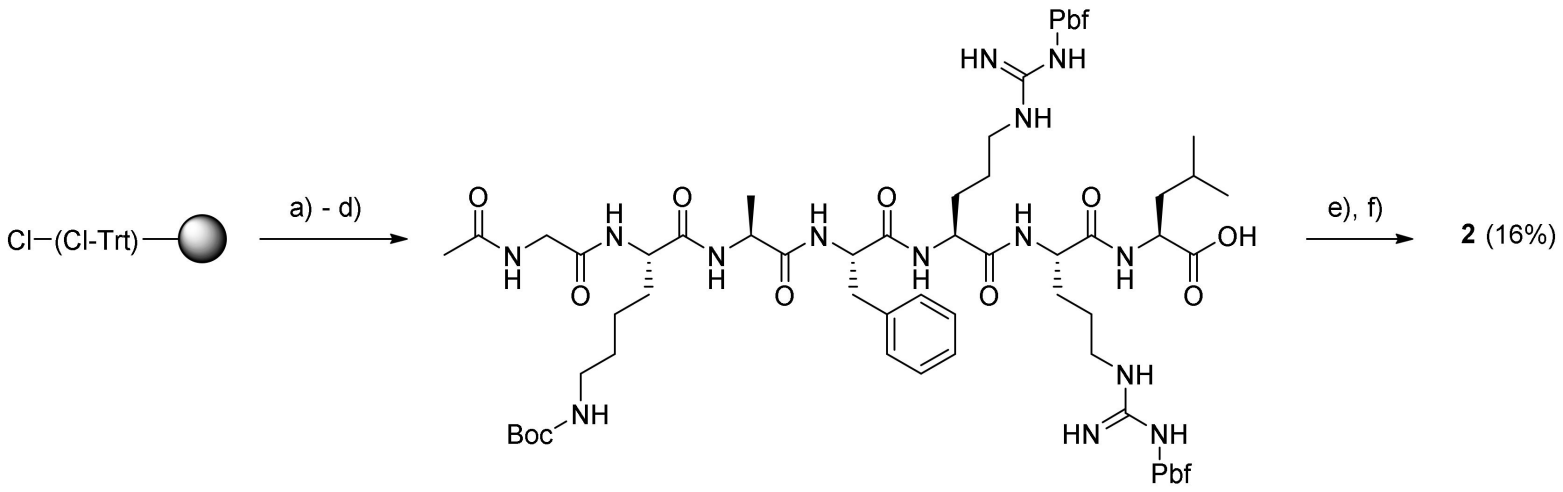
Synthesis of G114 (**2**). Reagents and conditions: (**a**) Fmoc–Leu–OH, DIPEA, DCM, rt, 3.5 h; (**b**) solid-phase peptide synthesis (SPPS) of non-acetylated peptide on a microwave peptide synthesizer; (**c**) Ac_2_O–DIPEA–NMP (1:2:3), rt, 2 × 10 min; (**d**) 20% HFIP–DCM, 3 × 30 min, rt; (**e**) **5**, PyBOP, DIPEA, DCM, DMF (few drops), rt, 13 h; (**f**) TFA (with a few drops of H_2_O added), rt, 24 h.

The synthesis of prodrugs **2** and **4** was undertaken by using a combination of solid-phase peptide synthesis (SPPS) and subsequent solution-phase conjugation to **5**. Thus, the peptide moieties of prodrugs **2** and **4** were assembled on Leu-preloaded 2-chlorotrityl resin by using a microwave-assisted peptide synthesizer (CEM Liberty Blue) operated at 45 °C in order to ensure a low degree of spontaneous cleavage during heating. Both peptides were modified at the N-terminus which, in the case of prodrug **2**, involved a standard acetylation procedure (Scheme 2), after which the resulting peptide was cleaved from the resin. As the peptide was considered to be the less expensive component, it was used in excess when coupled to **5** to provide the final prodrug, G-114 (**2**), after full deprotection with TFA (Supplementary Materials Figure S1–S7).

**Scheme 3:**
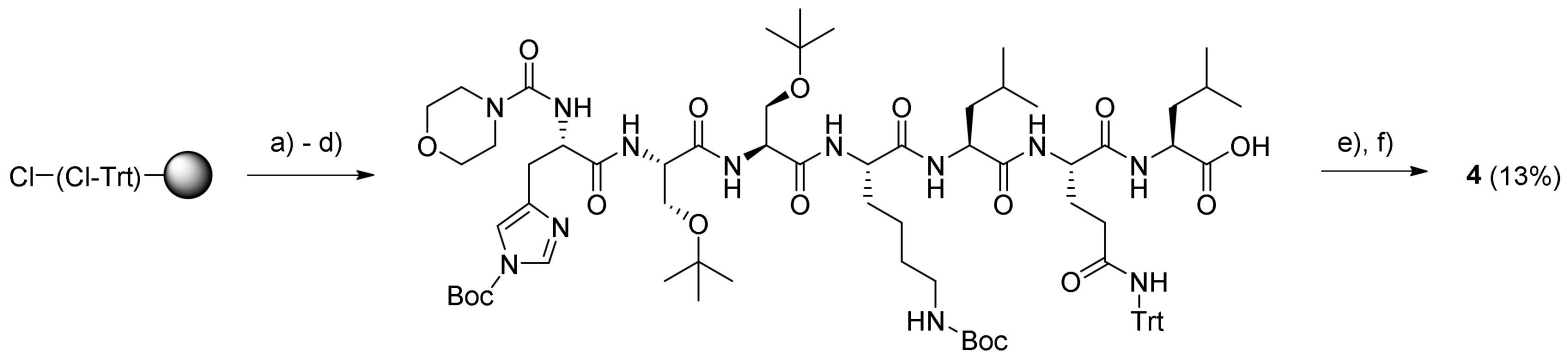
Synthesis of G115 (**4**). Reagents and conditions: (**a**) Fmoc–Leu–OH, DIPEA, DCM, rt, 3.5 h; (**b**) Microwave (MW)-assisted SPPS of peptide without N-terminal modification; (**c**) morpholine-4-carbonyl chloride–Et_3_N–NMP (1:4:20), 40 °C, 3 h; (**d**) 20% HFIP–DCM, 3 × 30 min, rt; (**e**) **5**, PyBOP, DIPEA, DCM, DMF (few drops), rt, 14 h; (**f**) TFA (with a few drops of H_2_O added), rt, 2 h.

The end modification of the peptide in prodrug **4** was less trivial as it required a novel protocol for the on-resin introduction of a morpholine-4-carbonyl moiety. After several attempts at room temperature, a procedure employing the mixture morpholine-4-carbonyl chloride–Et_3_N–NMP (1:4:20) and heating to 40 °C for 2 h, followed by a second treatment with new reagents for 1 h, provided a satisfactory conversion, as judged by the matrix-assisted laser desorption ionization-time of flight (MALDI-TOF) spectrum obtained for the reaction mixture. Subsequently, the protected peptide was cleaved from the resin and coupled with compound **5**, followed by deprotection with TFA to give the final prodrug, G-115 (**4**) (Scheme 3) (supplementary material Figure S8–S14).

**Scheme 4:**
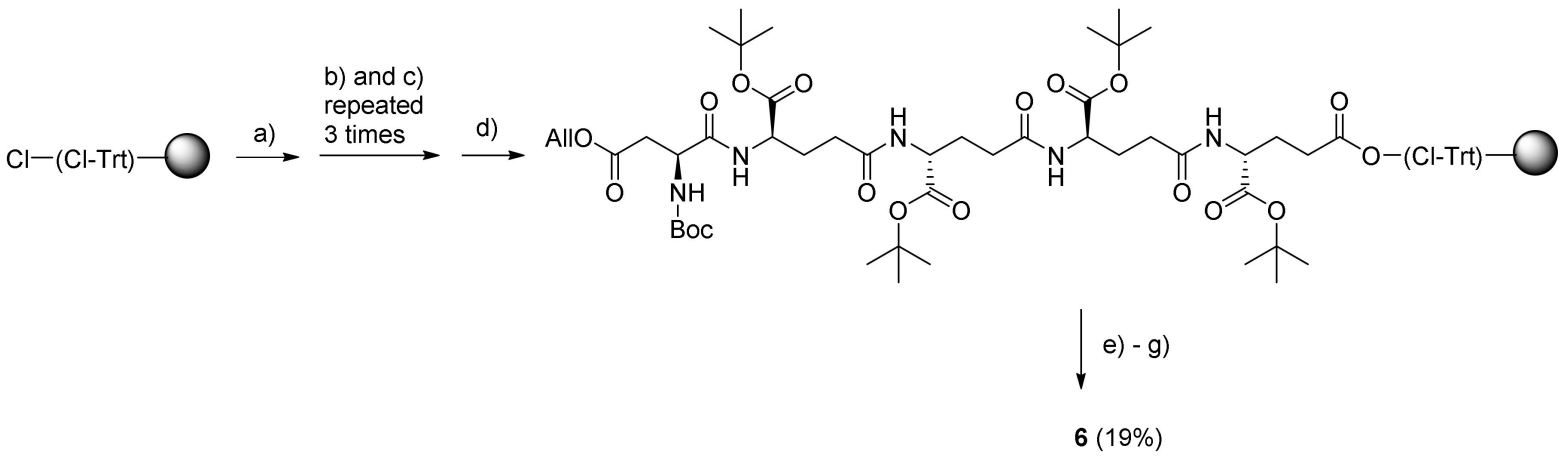
Continuous solid-phase synthesis (SPS) of Mipsagargin (**6**). Reagents and conditions: (**a**) Fmoc-Glu-O*t*Bu, DIPEA, DCM, 3 h; (**b**) Fmoc-Glu-O*t*Bu, PyBOP, DIPEA, DMF, 16 h; (**c**) 20% piperidine–DMF, 2 × 20 min; (**d**) Boc-Asp(All)-OH, PyBOP, DIPEA, DMF, 18 h; (**e**) Me_2_N**–**BH_3_ in DCM, Pd(PPh_3_)_4_ in DCM, 6 h; (**f**) **5**, PyBOP, DIPEA, DCM, 18 h; (**g**) TFA–DCM (1:1 with a few drops of H_2_O added), 1 h; all reactions were carried out at rt.

Previously, preparation of Mipsagargin (**6**) from the peptide EEEED and **5** was performed entirely by solution-phase chemistry [25], whereas, in the present work, a continuous solid-phase synthesis approach was utilized to obtain prodrug **6** (Scheme 4) (Supplementary Materials Figure S15–S20).

As the critical step was the final coupling of the large and bulky compound **5** to the resin-bound peptide, an excess of resin was employed for the initial loading of the C-terminal Fmoc-Glu-O*t*Bu building block in order to ensure it was only attached to readily accessible linker sites within the CTC resin particles. This was followed by three successive elongations with the same building block to assemble the (γ-Glu)_4_ peptide. Finally, the β-Asp residue was introduced by using Boc-Asp(All)-OH in order to achieve orthogonal protection of the Asp and Glu moieties, allowing for selective removal of the allyl group with Me_2_N–BH_3_ catalyzed by Pd(PPh_3_)_4_. Subsequently, the protected pentapeptide was coupled with thapsigargin derivative **5**. The cleavage from resin and removal of protecting groups were performed simultaneously by treatment with trifluoroacetic acid (TFA). This manual synthesis of the pentapeptide using a down-loading methodology proved to be both a convenient and efficient procedure when using a large portion of resin.

The purity of all target compounds (**2**, **4**, **6** and **8**) was verified by HPLC to be more than 95%, while their identities were confirmed by high-resolution mass spectrometry. Furthermore, these compounds were characterized by NMR spectroscopic methods (^1^H, ^13^C, COSY, HSQC, HMBC).

The target prodrug **2** has been cleaved efficiently by hK2 in a mouse model [26], while prodrug **4** has been cleaved efficiently by PSA and has, in vivo, been shown to inhibit the growth of prostate cancer in mice [19]. Moreover, prodrug **6** has been cleaved efficiently by PSMA and has been found to inhibit the growth of tumors in a mouse model [16] as well as prolonging disease stabilization in patients with liver cancer [22].

## 3. Materials and Methods

### 3.1. Starting Material, Reagents and Solvents

Thapsigargin (**1**) was obtained from the seeds of *Thapsia garganica* L., while Boc-12-aminododecanoate- (*O*-8)-debutanoyl-thapsigargin (i.e., the Boc-protected analogue of **5**) was prepared as earlier reported [27]. All Fmoc-protected standard amino acids, (2-(1*H*-benzotriazol-1-yl)-1,1,3,3-tetramethyluronium hexafluorophosphate (HBTU), diisopropylethylamin (DIPEA), 1,1,1,3,3,3-Hexafluoro-2-propanol (HFIP) and 2-chlorotrityl chloride (CTC) resin (loading: 1.0–1.6 mmol/g) were purchased from Iris Biotech GmbH (Marktredwitz, Germany). Borane dimethylamino complex and Pd(PPh_3_)_4_ were purchased from Sigma–Aldrich (St. Louis, MO, USA). Boc-Asp (All)-OH and Fmoc-Glu-O*t*Bu were purchased from Bachem AG (Bubendorf, Switzerland). All solvents and deprotection and cleavage reagents were of synthesis grade and were purchased from Iris Biotech GmbH (Marktredwitz, Germany). The solvents used for column chromatography, HPLC, HR-MS, and HR-MALDI-TOF were of HPLC grade and were purchased from VWR International (‎Radnor, PA, USA).

### 3.2. Solid-Phase Peptide Synthesis (SPPS)

Product **6** was synthesized by manual SPPS using a CTC resin preloaded with Fmoc–Glu–O*t*Bu. Peptides **2** and **4** were assembled on a CTC resin preloaded with Fmoc–Leu–OH by using a Liberty Blue^TM^ automated microwave peptide synthesizer (CEM Corp., Matthews, NC, USA) following an Fmoc/tBu protocol. In all cases, a CTC resin (loading: 1.0–1.6 mmol/g) was used as the solid phase. Couplings were performed with 0.2 M N^α^-Fmoc-protected amino acid building blocks (5 equiv; with acid-labile *t*Bu/Trt/Boc/Pbf as side chain protecting groups) in DMF in combination with 0.5 M HBTU (5.0 equiv) as the coupling reagent and 2 M DIPEA in NMP as the activator base. Fmoc deprotection was performed with 20% piperidine in DMF. The method used for peptide **2** synthesis was double coupling for 15 min at 45 °C with triple coupling of Arg. The method chosen for peptide **4** was single coupling for 15 min at 45 °C. Fmoc removal was performed with 20% piperidine in DMF at 45 °C for 30 and 180 s. Products were cleaved of the resin with 20% HFIP in DCM and then, amino acid protecting groups were deprotected with TFA (with a few drops of H_2_O added).

### 3.3. Compound Characterization

#### 3.3.1. HPLC

Water used for analytical and preparative HPLC was filtered through a 0.22 μm Millipore membrane filter. All final products were purified by reversed phase preparative HPLC on a Phenomenex Luna C18(2) column (250 × 21.2 mm; 5 µm particle size) on a Shimadzu Prominence system using an aqueous MeCN gradient with 0.1% TFA added (eluent A: 5:95 MeCN–H_2_O + 0.1% TFA, eluent B: 95:5 MeCN–H_2_O + 0.1% TFA). Elution was performed with linear gradients for 20 min at a flow rate of 20 mL/min with UV detection at λ = 220 nm. Purity was determined by analytical HPLC on a Phenomenex Luna C18(2) HTS column (100 mm × 3.0 mm; 2.5 µm particle size) using a Shimadzu Prominence and Shimadzu Nexera system with the same eluents as those used for preparative HPLC and a flow rate of 0.5 mL/min. All tested compounds had a purity of at least 95%.

#### 3.3.2. NMR Spectroscopy

^1^H and ^13^C nuclear magnetic resonance (NMR) spectra were recorded on a 600 MHz Bruker Avance III HD spectrometer equipped with a cryogenically-cooled 5 mm dual probe or on a 400 MHz Bruker Ascend spectrometer. Samples were dissolved in methanol-*d*_4_ (Cambridge Isotope Laboratories) and analyzed at 300 K. The residual solvent peak was used as an internal reference (methanol-*d*_4_: δ_C_ = 49.00; δ_H_ = 3.31). Coupling constants (*J* values) are given in hertz (Hz). Multiplicities were reported as follows: singlet (s), doublet (d), triplet (t), quartet (q), and multiplet (m). Thapsigargin guaianolide skeleton was numbered as depicted in Figure 1 (**1**), and ester substituents were labeled as follows: Ang for the angeloyl moiety at *O*-3, Oct for the octanoyl moiety at *O*-2, and 12-AD for the 12-Aminododecanoate moiety at *O*-8.

#### 3.3.3. Mass Spectrometry

High-resolution mass spectra were recorded on a Bruker MicroTOF-Q LC mass spectrometer equipped with an electrospray ionization source or on a Quadropole MS detector or via MALDI-TOF on a Bruker SolariX XR in MALDI mode. The analyses were performed in positive ionization mode to give peaks of [M + nH]^n+^.

### 3.4. Synthesis and Purification of Compounds ***2***, ***4***, ***5***, ***6***, and ***8***

#### 3.4.1. Ac-Gly-Lys-Ala-Phe-Arg-Arg-Leu-12-Aminododecanoate-(*O*-8)-Debutanoyl-Thapsigargin (**2**)

Loading of the C-terminal residue was performed by swelling 2-chlorotrityl chloride (CTC) resin (1.6 mmol/g; 1.0 g) in DCM (4 mL), followed by the addition of Fmoc-Leu-OH (564 mg; 1.6 mmol) dissolved in DCM (4 mL) and DIPEA (2.7 mL; 16 mmol). The mixture was shaken for 3.5 h at rt, and then the resin was washed with DCM, capped by treatment with DIPEA–MeOH–DCM 5:15:80 (8 mL; 2 × 5 min) and subsequently, washed with DMF, MeOH, and DCM (each with 3 × 10 mL for 3 min). Test cleavage with 20% HFIP–DCM of a small amount of dry loaded resin, followed by careful evaporation and weighing of the amount of cleaved Fmoc-Leu-OH showed a loading of ~0.9 mmol/g.

The peptide Ac-GKAFRRL-OH was synthesized on a Liberty Blue^TM^ microwave peptide synthesizer starting from the above Fmoc-Leu-*O*-2-CTC resin (0.9 mmol/g; 110 mg; 0.1 mmol) swelled in DMF (5 mL). Calculated amounts of N^α^-Fmoc protected amino acids in 0.2 M solution (added as volumes corresponding to 5 equivalents): Fmoc-Gly-OH (0.33 g; 11 mL DMF), Fmoc-Lys(Boc)-OH (0.52 g; 11 mL DMF), Fmoc-Ala-OH (0.35 g; 11 mL DMF), Fmoc-Phe-OH (0.43 g; 11 mL DMF), and Fmoc-Arg(Pbf)-OH (2.08 g; 32 mL DMF). The conditions for chain elongation were double coupling at 45 °C for 15 min, while triple coupling at 45 °C during 15 min was needed for Arg; Fmoc removal was performed with 20% piperidine in DMF at 45 °C for 30 s and 180 s.

The resulting Gly-Lys-Ala-Phe-Arg-Arg-Leu-*O*-(Cl-Trt)-resin was washed twice with both DMF and DCM, and was then acetylated with Ac_2_O–DIPEA–NMP (1:2:3, 6 mL; 2 × 5 min). The resulting resin-bound peptide was washed with DMF, MeOH, and DCM (each 3 × 8 mL for 3 min) and cleaved from the resin with 20% HFIP–DCM. The drained cleavage solution was concentrated to give the protected peptide as a gel. To a solution of the residue (345 mg; 0.23 mmol) in DCM (1.5 mL) and PyBOP (48 mg; 0.092 mmol) in DMF (0.4 mL) and DIPEA (27 µL; 0.132 mmol) were added. The mixture was stirred for 20 min to pre-activate the carboxylic acid, and then a solution of compound **5** (60 mg; 0.077 mmol) in DCM (1.5 mL) was added. The mixture was stirred under argon for 14 h at rt, and the resulting reaction mixture was concentrated in vacuo. The obtained residue was deprotected with TFA (3 mL with four drops of H_2_O added) for 2 h at rt. The reaction mixture was evaporated, and the residue was subjected to purification by preparative HPLC using the gradient 20% → 80% B over 20 min. The purity of the product (retention time: 13.0–13.7 min) was estimated by analytical UHPLC to be >98% using the gradient 20% → 100% B for 10 min (retention time: 7.7 min). Upon concentration, the product was dissolved in dioxane–H_2_O and freeze-dried to provide prodrug **2** (20 mg; 15.7%) as a white solid. Characterization of prodrug **2**: ^1^H NMR (400 MHz, methanol-*d*_4_) δ ppm = 7.32–7.28 (m, 2 H, H_Phe_-ε), 7.27–7.25 (m, 2 H, H_Phe_-δ), 7.22 (m, 1 H, H_Phe_-ζ), 6.18 (qq, *J* = 7.2, 1.5 Hz, 1 H, H_Ang_-3), 5.71 (m, 1 H, H-6), 5.68 (m, 1 H, H-3), 5.59 (t, *J* = 3.7 Hz, 1 H, H-8), 5.52 (t, *J* = 2.9 Hz, 1 H, H-2), 4.47 (m, 1 H, H_Phe_-α), 4.36 (m, 1 H, H-1), 4.33 (m, 1 H, H_Lys_-α), 4.30 (m, 1 H, H_Arg_-α), 4.24 (m, 1 H, H_Arg_-α), 4.20 (m, 1 H, H_Leu_-α), 4.17 (m, 1 H, H_Ala_-α), 3.97–3.83 (m, 2 H, 2 H_Gly_-α), 3.27–3.18 (m, 6 H, 2 H_Arg_-δ and H_Phe_-β), 3.17–3.10 (m, 2 H, H_Lys_-ε), 3.02 (m, 1 H, H-9_a_), 2.95 (m, 2 H, H_12-AD_-12), 2.37 (m, 2 H, H_oct_-2), 2.32 (m, 1 H, H-9_b_), 2.29 (m, 2 H, H_12-AD_-2), 2.02–1.98 (m, 3 H, H_Ang_-4), 2.00 (s, 3 H, -(C=O)CH_3_), 1.93 (m, 3 H, CH_3_ from C_Ang_-2), 1.89 (s, 3 H, Ac_Tg_), 1.86 (m, 3 H, H-15), 1.85–1.80 (m, 2 H, H_Arg_-β), 1.80–1.74 (m, 2 H, H_Lys_-β), 1.74–1.71 (m, 2 H, H_Arg_-β), 1.71 (m, 1 H, H_Leu_-γ), 1.70–1.67 (m, 4 H, 2 H_Arg_-γ), 1.66 (m, 2 H, H_Leu_-β), 1.64 (m, 2 H, H_Lys_-δ), 1.62 (m, 2 H, H_oct_-3), 1.61–1.56 (m, 2 H, H_12-AD_-3), 1.54–1.49 (m, 2 H, H_12-AD_-11), 1.49–1.44 (m, 2 H, H_Lys_-γ), 1.42 (s, 3 H, H-14), 1.37 (s, 3 H, H-13), 1.36–1.28 (m, 25H, H_12-AD_-4 − 10, H_oct_-4 − 7, H_Ala_-β), 0.97*–*0.92 (m, 6 H, 2 H_Leu_-δ), 0.92–0.89 (m, 3 H, H_oct_-8); ^13^C NMR (400 MHz, methanol-*d*_4_) δ ppm = 176.72 (1 C, C-12), 174.53 (1 C, C_Ala_-1), 173.51 (1 C, C_Leu_-1), 172.98 (1 C, C_Phe_-1), 172.89 (2 C, C_Ac capped_-1, C-1 Lys), 172.84 (1 C, C_oct_-1), 172.59 (1 C, C_Arg_-1), 172.39 (1 C, C_Arg_-1), 172.35 (1 C, C_12-AD_-1), 171.65 (1 C, C_Gly_-1), 170.42 (1 C, C_Ac Tg_-1), 167.18 (1 C, C_Ang_-1), 157.25 (2 C, 2 C_Arg_-6), 139.66 (1 C, C-4), 138.07 (1 C, C_Ang_-3), 131.96 (1 C, C-5), 128.85 (1 C, C_Ang_-2), 128.83 (2 C, C_Phe_-6 + 8), 128.19 (2 C, C_Phe_-5 + 9), 127.43 (1 C, C_Phe_-4), 126.53 (1 C, C_Phe_-7), 84.52 (1 C, C-3), 84.30 (1 C, C-10), 78.09 (1 C, C-11), 78.05 (1 C, C-7), 77.92 (1 C, C-2), 76.62 (1 C, C-6), 66.04 (1 C, C-8), 57.68 (1 C, C-1), 55.76 (1 C, C_Phe_-2), 54.60 (1 C, C_Leu_-2), 53.44 (2 C, 2 C_Arg_-2), 52.14 (1 C, C_Lys_-2), 50.47 (1 C, C_Lys_-2), 42.92 (1 C, C_Gly_-2), 40.55 (3 C, C_Leu_-3 Leu + 2 C_Arg_-5), 39.07 (2 C, C_12-AD_-12 and C_Lys_-6), 38.02 (1 C, C-9), 34.14 (1 C, C_12-AD_-2), 33.78 (1 C, C_oct_-2), 31.45 (1 C, C_oct_-7), 30.33 (1 C, C_Lys_-3), 29.31–28.68 (9 C, C_12-AD_-4 − 9 and 11, C_oct_-4 − 5), 27.84 (2 C, 2 C_Arg_-3), 26.73 (2 C, 2 C_Arg_-4), 26.53 (1 C, C_12-AD_-11), 24.84 (1 C, C_Lys_-5), 24.57 (2 C, C_oct_-3 and C_12-AD_-3), 24.18 (1 C, C_Leu_-4), 22.25 (1 C, C_oct_-6), 22.16 (2 C, C_Lys_-3 and C_Leu_-5_a_), 21.95 (1 C, C-14), 21.29 (1 C, C_Ac Tg_-2 Tg), 21.14 (1 C, CH_3_ in Ac-2), 20.42 (1 C, C_Leu_-5_b_), 19.35 (1 C, CH_3_ from C_Ang_-2), 15.65 (1 C, C_Ala_-3), 14.67 (1 C, C_Ang_-4), 14.46 (1 C, C-13), 13.02 (1 C, C_oct_-8), 11.58 (1 C, C-15); HR-MALDI-TOF [C_82_H_133_N_15_O_20_ + H]^+^: *m*/*z*: 1648.9924. Found 1648.9929.

#### 3.4.2. Morpholine-4-Carbonyl-His-Ser-Ser-Lys-Leu-Gln-Leu-12-Aminododecanoate-(*O*-8)- Debutanoyl-Thapsigargin (**4**)

Synthesis of the HSSKLQL peptide was performed on a Liberty Blue^TM^ microwave peptide synthesizer starting from the above Fmoc-Leu-*O*-2-CTC resin (0.9 mmol/g; 110 mg; 0.1 mmol) swelled in DMF (5 mL). The calculated amounts of N^α^-Fmoc protected amino acids in 0.2 M solution (added as volumes corresponding to 5 equivalents) were: Fmoc-His(Trt)-OH (0.38 g; 6 mL DMF), Fmoc-Ser(*t*Bu)-OH (0.43 g; 11 mL DMF), Fmoc-Lys(Boc)-OH (0.29 g; 6 mL DMF), Fmoc-Leu-OH (0.22 g; 6 mL DMF), and Fmoc-Gln(Trt)-OH (0.37 g; 6 mL DMF). The conditions for chain elongation were single coupling at 45 °C for 15 min; Fmoc removal was performed with 20% piperidine in DMF at 45 °C for 30 s and 180 s.

The resulting protected His-Ser-Ser-Lys-Leu-Gln-Leu-*O*-(Cl-Trt) resin was placed in two reaction vessels, and then, the resin was washed twice with both DMF and DCM. Solutions of morpholine-4-carbonyl chloride–Et_3_N–NMP (1:4:20; 8 mL) were added to the two resin batches, which then were heated to 40 °C for 2 h. The resin was washed with DCM, and the procedure was repeated for 1 h with fresh reagents, followed by washing with DMF, MeOH, and DCM (each 3 × 8 mL for 3 min). The product was cleaved from the resin with 20% HFIP–DCM, and the concentration of the drained cleavage mixture gave the protected peptide as a gel (220 mg). A portion of this residue (165 mg) was dissolved in DCM (1.5 mL), and then, PyBOP (41 mg; 0.078 mmol) and DIPEA (23 µL; 0.178 mmol) were added. The mixture was stirred for 20 min to pre-activate the carboxylic acid, followed by the addition of compound **5** (52 mg; 0.067 mmol) dissolved in DCM (1.5 mL). The mixture was stirred under argon for 14 h at rt. The resulting reaction mixture was concentrated in vacuo, and the residue was deprotected with TFA (3 mL; with four drops of H_2_O added) for 2 h at rt. The drained cleavage mixture was evaporated, and the residue was subjected to purification by preparative HPLC using the gradient 20% → 80% B over 20 min. The purity of the product (retention time: 16.7–17.2 min) was determined by analytical UHPLC (>99%) using the gradient 20% → 100% B for 10 min (retention time: 8.2 min). Upon concentration, the resulting residue was dissolved in dioxane–H_2_O and freeze-dried to provide prodrug **4** (15 mg; 13.4%) as a white solid. Characterization of prodrug **4**: ^1^H NMR (400 MHz, methanol-*d*_4_) δ ppm = 8.82 (s, 1 H, H_His_-ε_1_), 7.39 (s, 1 H, H_His_-δ_2_), 6.19 (qq, *J* = 7.2, 1.5 Hz, 1 H, H_Ang_-3), 5.71 (m, 1 H, H-6), 5.68 (m, 1 H, H-3), 5.60 (t, *J* = 3.7 Hz, 1 H, H-8), 5.53 (t, *J* = 2.9 Hz, 1 H, H-2), 4.61 (m, 1 H, H_His_-α), 4.52–4.46 (m, 2 H, 2 H_Ser_-α), 4.36 (m, 1 H, H-1), 4.32 (m, 1 H, H_Lys_-α), 4.3 (m, 1 H, H_Gln_-α), 4.29–4.24 (m, 2 H, 2 H_Leu_-α), 4.02–3.91 (m, 2 H, 2 H_Ser a_-β), 3.87–3.80 (m, 2 H, 2 H_Ser b_-β), 3.66 (t, *J* = 4.4 Hz, 4 H, H_Morph_-2 and 6), 3.41 (t, *J* = 5.1 Hz, 4 H, H_Morph_-3 and 5), 3.30 (m, 1 H, H_His a_-β), 3.20 (m, 2 H, H_12-AD_-12), 3.15 (m, 1 H, H_His b_-β), 3.01 (m, 1 H, H-9_a_), 2.96 (m, 2 H, H_Lys_-ε), 2.37 (m, 2 H, H_oct_-2), 2.35 (m, 2 H, H_Gln_-γ), 2.33 (m, 1 H, H-9_b_), 2.30 (m, 2 H, H_12-AD_-2), 2.07–2.15 (m, 2 H, H_Gln_-β), 2.00 (dq, *J* = 5.9. 1.0 Hz, 2 H, H_Ang_-4), 1.94 (m, 2 H, CH_3_ from C_Ang_-2), 1.91 (m, 1 H, H_Lys a_-β), 1.90 (s, 3 H, Ac), 1.87 (s, 3 H, H-15), 1.80 (m, 1 H, H_Lys b_-β), 1.75–1.71 (m, 2 H, 2 H_Leu_-γ), 1.70 (m, 2 H, H_Lys_-δ), 1.69–1.66 (m, 4 H, 2 H_Leu_-β), 1.65 (m, 2 H, H_oct_-3), 1.63 (m, 2 H, H_12-AD_-3), 1.54 (m, 2 H, H_12-AD_-11), 1.50 (m, 2 H, H_Lys_-γ), 1.42 (s, 3 H, H-14), 1.37 (s, 3 H, H-13), 1.36–1.29 (m, 22 H, H_12-AD_-4 − 10, H_oct_-4 − 7), 0.97 (m, 6 H, 2 H_Leu_-δ) 0.93 (m, 6 H, 2 H_Leu_-δ), 0.91 (m, 3 H, H_oct_-8); ^13^C NMR (400 MHz, methanol-*d*_4_) δ ppm = 176.72 (1 C, C-12), 173.96 (1 C, C_Leu_-1), 173.61 (1 C, C_Gln_-1), 173.51 (1 C, C_Leu_-1), 173.15 (1 C, C_Gln_-5), 172.89 (1 C, C_12-AD_-1), 172.45 (1 C, C_Lys_-1), 172.35 (1 C, C_oct_-1), 172.22 (1 C, C_His_-1), 171.70 (2 C, 2 C_Ser_-1), 170.43 (1 C, C_Ac_-1), 167.18 (1 C, C_Ang_-1), 157.99 (1 C, Morph. carbonyl), 139.66 (1 C, C-5), 138.07 (1 C, C_Ang_-3), 133.60 (1 C, C_His_-6) 131.98 (1 C,C-4), 129.94 (1 C, C_Ang_-2), 127.43 (1 C, C_His_-4), 117.12 (1 C, C_His_-5), 84.52 (1 C, C-3), 84.30 (1 C, C-10), 78.09 (1 C, C-11), 78.04 (1 C, C-7), 77.93 (1 C, C-2), 76.62 (1 C, C-6), 66.12 (2 C, C_Morph_-3 + 5), 66.04 (1 C, C-8), 61.45 (1 C, C_Ser_-3), 61.30 (1 C, C_Ser_-3), 57.67 (1 C, C-1), 55.98 (1 C, C_Ser_-2), 55.67 (1 C, C_Ser_-2), 54.50 (1 C, C_Leu_-2), 54.40 (1 C, C_Leu_-2), 54.20 (1 C, C_His_-2), 53.59 (1 C, C_Gln_-2), 52.14 (1 C, C_Lys_-2), 43.93 (2 C, C_Morph_-3 + 5), 40.42 (1 C, C_Leu_-3), 39.61 (1 C, C_Leu_-3), 39.10 (2 C, C_12-AD_-12 + C_Lys_-6), 38.02 (1 C, C-9), 34.14 (1 C, C_12-AD_-2), 33.78 (1 C,C_oct_-2), 31.45 (1 C, C_oct_-7), 31.40 (1 C, C_Gln_-4), 30.08 (1 C, C_Lys_-3), 29.28–28.69 (9 C, C_12-AD_-4 − 9 + 11, C_oct_-4 − 5), 27.00 (1 C, C_Gln_-3), 26.70 (1 C, C_12-AD_-10), 26.54 (1 C, C_His_-3), 26.49 (1 C, C_Lys_-5), 24.60 (1 C, C_12-AD_-3), 24.57 (2 C, 2 C_Leu_-4), 24.19 (1 C, C_oct_-4), 22.25 (2 C, 2 C_Leu_-5), 22.17 (1 C, C_oct_-6), 22.09 (1 C, C_Lys_-4), 22.01 (1 C, C-14), 20.31 (2 C, 2 C_Leu_-5) 19.35 (1 C, CH_3_ from C_Ang_-2), 14.67 (1 C, C_Ang_-4), 14.47 (1 C, C-13), 13.02 (1 C, C_oct_-8), 11.58 (1 C, C-15); HR-MALDI-TOF: [C_82_H_133_N_13_O_2_ + H]^+^ *m*/*z*: 1683.9586. Found 1684.9673.

#### 3.4.3. 12-Aminododecanoate-(*O*-8)-Debutanoyl-Thapsigargin (**5**)

To a solution of Boc-12-aminododecanoate-(*O*-8)-debutanoyl-thapsigargin (150 mg; 0.171 mmol) [27] in DCM (0.5 mL) TFA (2 mL) was added, together with 4 drops of H_2_O. The mixture was stirred at rt for 90 min. The reaction mixture was concentrated, and the was residue purified on a silica column (DCM–MeOH 10:1) to provide **5** (120 mg; 90%) as a yellowish solid. ^1^H NMR, ^13^C NMR and HR-MS were as reported [27].

#### 3.4.4. (γ-Glu-)_4_-β-Asp-12-Aminododecanoate-(*O*-8)-Debutanoyl-Thapsigargin (**6**)

In a glass vessel (300 mL) for manual SPPS (Peptides International, Louisville, KY, USA) 2-chlorotrityl chloride resin (1.6 mmol/g; 3.125 g; 5 mmol) was swelled in DCM (8 mL), and upon draining, a solution of Fmoc-Glu-O*t*Bu (425 mg; 1 mmol) and DIPEA (2.1 mL; 12 mmol) in DCM (15 mL) was added to the reaction vessel. After shaking for 3 h at rt, the resin was drained, washed with DCM, and then capped with DIPEA–MeOH–DCM 5:15:80 (8 mL; 2 × 5 min), and finally, it was washed with DMF, MeOH, and DCM (each 3 times for 3 min). The residual solvent was removed on a freeze-dryer. Test cleavage of a dry sample of the loaded resin showed a loading of ca. 0.30 mmol/g.

By using a large syringe (with a polypropylene filter bottom), the peptide was assembled on the above resin preloaded with Fmoc-Glu-O*t*Bu (0.304 mmol/g; 1.5 g), starting with three sequential coupling cycles, each comprising Fmoc removal with 20% piperidine–DMF (8 mL; 2 × 20 min), washing of the resin with DMF, MeOH, and DCM (each 3 times for 3 min), the addition of DMF (3 mL) and DCM (2 mL), and the addition of a solution of Fmoc-Glu-O*t*Bu (390 mg; 0.95 mmol) pre-activated (10 min) with PyBOP (711 mg; 1.37 mmol) and DIPEA (0.475 mL; 2.72 mmol) in DMF (3 mL). Each coupling was performed under shaking for 5 h at rt, and then the resin was washed with DMF, MeOH, and DCM (each 3 times for 3 min). Boc-Asp(All)-OH (350 mg; 1.28 mmol) was coupled by using the same conditions. Allyl deprotection was performed on-resin by adding DCM (5 mL) followed by a solution of Me_2_N**·**BH_3_ in DCM (2 mL) and 0.02 M Pd(PPh_3_)_4_ (185 mg) in DCM (8 mL). The mixture was shaken for 6 h under argon at rt, after which the resin was washed with DMF, MeOH, and DCM (each 3 times for 3 min).

DCM (4 mL) was added to part of the resin-bound peptide (0.75 g), and a solution of PyBOP (356 mg; 0.68 mmol) and DIPEA (0.24 mL; 1.36 mmol) in DCM (4 mL) was added to pre-activate the carboxylic acid for 20 min. A solution of **5** (354 mg; 0.46 mmol) in DCM (2 mL) was added, and then the mixture was shaken for 10 h at rt. Finally, the resin was drained and washed with DMF, MeOH, and DCM (each 3 times for 3 min), and the protected conjugate was cleaved off the resin and protecting groups were removed by treatment with TFA (4 mL), DCM (0.5 mL), and H_2_O (4 drops) for 2.5 h at rt. The concentration of the reaction mixture afforded a crude product, which was purified by preparative HPLC using a gradient of 30% → 100% B (20 min; retention time 13.2–13.7 min) to give the desired product, which, as assessed by analytical UHPLC, had a purity >99% when using the same gradient 30% → 100% B (10 min; retention time 7.4 min). This fraction was evaporated to give a residue, which was dissolved in dioxane–H_2_O and freeze-dried to give prodrug **6** (60 mg; 18.7%) as a white solid. Characterization of prodrug **6**: ^1^H NMR (600 MHz, methanol-*d*_4_) δ ppm = 6.13 (qq, *J* = 7.2, 1.5 Hz, 1 H, H_Ang_-3), 5.65 (m, 1 H, H-6), 5.62 (m, 1 H, H-3), 5.53 (t, *J* = 3.7 Hz, 1 H, H-8), 5.46 (t, *J* = 2.9 Hz, 1 H, H-2), 4.45–4.37 (m, 4 H, H_Glu_-α), 4.30 (m, 1 H, H-1), 4.26 (m, 1 H, H_Asp_-α), 3.15 (sxt, *J* = 6.6 Hz, 2 H, H_12-AD_-12), 2.93 (dd, *J* = 14.5, 3.5 Hz, 1 H, H_a_-9), 2.87 (dd, *J* = 16.5, 4.4 Hz, 1 H, H_Asp a_-3), 2.71 (dd, *J* = 9.0, 16.7 Hz, 1 H, H_Asp b_-3), 2.41–2.32 (m, 8 H, H-γ Glu), 2.32–2.26 (m, 4 H, H_oct_-2 and H_12-AD_-2), 2.25 (m, 1 H, H_b_-9), 2.24–2.10 (m, 6 H, H_Glu2-Glu4_-β), 1.94 (dq, *J* = 5.9, 1.0 Hz, 3 H, H_Ang_-4), 1.91 (dd, *J* = 8.8, 6.6 Hz, 2 H, H_Glu1_-β), 1.88 (t, *J* = 1.5 Hz, 3 H, CH_3_ from C_Ang_-2), 1.83 (m, 3 H, Ac), 1.81 (s, 3 H, H-15), 1.62–1.52 (m, 4 H, H_oct_-3 and H_12-AD_-3), 1.46 (m, 2 H, H_12-AD_-11), 1.36 (s, 3 H, H-14), 1.31 (s, 3 H, H-13), 1.30–1.23 (m, 22 H, H_12-AD_-4 − 10, H_oct_-4 − 7), 0.86 (m, 3 H, H_oct_-8); ^13^C NMR (600 MHz, methanol-*d*_4_) δ ppm = 176.71 (1 C, C-12) 175.01 (1 C, C_Glu1_-5), 173.68–173.60 (6 C, C_Glu1-Glu4_ -1 and C_Glu2-Glu3_-5), 173.43 (1 C, C_Glu4_-5), 172.78 (1 C, C_oct_-1), 172.35 (1 C, C_12-AD_-1), 170.43 (1 C, C-1 Ac), 169.22 (1 C, C_Asp_-4), 168.27 (1 C, C_Asp_-1), 167.18 (1 C, C_Ang_-1), 139.65 (1 C, C-5), 138.08 (1 C, C_Ang_-3), 131.95 (1 C, C-4), 127.42 (1 C, C_Ang_-2), 84.52 (1 C, C-10), 84.30 (1 C, C-3), 78.08 (1 C, C-11), 78.04 (1 C, C-7), 77.92 (1 C, C-2), 76.62 (1 C, C-6), 66.04 (1 C, C-8), 57.66 (1 C, C-1), 52.01 (1 C, C_Glu1_-2), 51.79 (1 C, C_Glu2_-2), 51.71 (1 C, C_Glu3_-2), 51.67 (1 C, C_Glu4_-2), 50.10 (1 C, C_Asp_-2), 39.28 (1 C, C_12-AD_-12), 38.01 (1 C, C-9), 35.12 (1 C, C_Asp_-3), 34.14 (1 C, C_12-AD_-2), 33.78 (1 C, C_oct_-2), 31.56–31.51 (3 C, C_Glu2-Glu4_-4), 31.45 (1 C, C_oct_-7), 29.87 (1 C, C_Glu1_-4), 29.30–28.65 (9 C, C_12-AD_-4 − 9 and 11, C_oct_-4 − 5), 27.07 (1 C, C_Glu4_-3), 26.91 (1 C, C_Glu3_-3), 26.86 (1 C, C_Glu2_-3), 26.66 (1 C, C_12-AD_-10), 26.47 (1 C, C_Glu4_-3), 24.56 (1 C, C_oct_-3), 24.18 (1 C, C_12-AD_-3), 22.26 (1 C, C_oct_-6), 21.96 (1 C, C-14), 21.30 (1 C, C-2 Ac), 19.36 (1 C, CH_3_ from C_Ang_-2), 14.68 (1 C, C_Ang_-4), 14.45 (1 C, C-13), 13.04 (1 C, C-8), 11.57 (1 C, C-15); HR-MS was as reported [25].

#### 3.4.5. Ac-Asp-12-Aminododecanoate-(*O*-8)-Debutanoyl-Thapsigargin (**8**)

Fmoc-OSu (6.55 g; 21.9 mmol) and NaHCO_3_ (2.39 g; 28.5 mmol) were added to a solution of 12-aminododecanoic acid (4.71 g; 21.9 mmol) in acetone (90 mL) and water (90 mL), and then the mixture was stirred at rt for 20 h. The reaction mixture was quenched with concentrated HCl until pH 4–5, and the resulting precipitate was extracted into with EtOAc (3 × 50 mL). The resulting combined organic phases were dried with Na_2_SO_4_, filtered, and evaporated. The crude product was purified on a silica column (Hexane–EtOAc 3:1) to provide Fmoc-12-aminododecanoic acid (4.20 g; 43%) as a white solid. ^1^H NMR and ^13^C NMR were as reported [28].

2-Chlorotrityl resin (2.58 g; loading: 1.6 mmol/g) was swelled in DCM (5 mL) in a teflon reaction vessel fitted with a polypropylene (PP) filter, and a solution of Fmoc-12-aminododecanoic acid (0.60 g; 1.37 mmol) and DIPEA (2.38 ml; 13.7 mmol) in DCM (3 mL) was added to the resin. The mixture was shaken for 3 h at rt. Then, the resin was drained and washed twice with DCM followed by capping with DIPEA–MeOH–DCM 5:15:80 (8 mL; 2 × 5 min). The resin was washed successively with DMF, MeOH, and DCM (each 3 times with 8 mL for 3 min); the residual solvent was removed on a freeze-dryer. Test cleavage of the dry preloaded resin with 20% HFIP in DCM showed a loading of ~0.48 mmol/g.

The Fmoc protecting group was removed with 20% piperidine–DMF (8 mL; 2 × 20 min), followed by washing with DMF, MeOH, and DCM (each 3 times as above). A solution of Fmoc-Asp-O*t*Bu (587 mg; 1.42 mmol), PyBOP (743 mg; 1.42 mmol) and DIPEA (0.25 mL; 2.62 mmol) dissolved in DMF (6 mL) was added to the resin-bound 12-aminododecanoic acid (1 g, 0.48 mmol loading). The mixture was shaken for 16 h at rt, after which the resin was washed with DMF, MeOH, and DCM (each 3 times for 3 min). Then, resin **9** was dried, and a portion (502 mg) was divided into two vessels where it was Fmoc-deprotected with 20% piperidine–DMF (8 mL; 2 × 20 min), washed twice with DMF and DCM, and finally, acetylated with Ac_2_O–DIPEA–NMP (1:2:3; 6 mL; 2 × 5 min). After a final standard washing of the resin, it was subjected to cleavage with 20% HFIP–DCM (3 × 6 mL; each time for 30 min), and the resulting mixture was concentrated to give intermediate **10** (110 mg; 0.22 mmol; 90%). 

Intermediate **10** (50 mg; 0.13 mmol) was coupled to **11** (50 mg; 0.086 mmol) using EDC (26 mg; 0.14 mmol) and DMAP (6 mg; 0.05 mmol) in DCM (2 mL). The mixture was stirred for 23 h under argon at rt. The resulting reaction mixture was evaporated and filtered through a silica column using DCM–MeOH 30:1 as the eluent. Finally, deprotection of the crude *tert*-butyl ester was performed with TFA (3 mL with 4 drops of water added) for 2 h at rt. The resulting crude product was purified by preparative HPLC with a gradient of 50% → 100% B for 20 min to give **8** (retention time 18-18.5 min). Upon evaporation, the product was dissolved in dioxane–H_2_O and freeze-dried to provide compound **8** (29 mg; 36%) as a white solid. An analytical UHPLC with the gradient 50% → 100% B for 10 min: showed a retention time of 8.4 min and a purity of >99%. Characterization of compound **8**: ^1^H NMR (600 MHz, methanol-*d*_4_) δ ppm = 6.18 (qd, *J* = 7.2, 1.5 Hz, 1 H, H_Ang_-3), 5.70 (m, 1 H, H-6), 5.67 (m, 1 H, H-3), 5.59 (t, *J* = 3.7 Hz, 1 H, H-8), 5.52 (t, *J* = 2.9 Hz, 1 H, H-2), 4.73 (dt, *J* = 7.0, 5.5 Hz, 1 H, H_asp_-α), 4.36 (m, 1 H, H-1), 3.16 (dt, *J* = 7.2, 1.5 Hz, 2 H, H_12-AD_-12), 2.99 (dd, *J* = 14.7, 3.7 Hz, 1 H, H_a_-9), 2.66–2.78 (m, 2 H, H_Asp_-β), 2.38 (m, 2H, CH_oct-_2), 2.32 (m, 2 H, CH_12-AD_-2), 2.29 (dd, *J* = 11.4, 3.3 Hz, 1 H, H_b_-9), 2.00 (dq, 5.9, 1.0 Hz, 3 H, H_Ang_-4), 1.99 (s, 3 H, Ac_Asp_), 1.93 (t, *J* = 1.5 Hz, 3 H, CH_3_ from C_Ang_-2), 1.89 (s, 3 H, Ac_Tg_), 1.86 (s, 3 H, H-15), 1.64 (m, 2H, CH_oct_-3), 1.61 (m, 2H, CH_12-AD_-3), 1.50 (m, 2 H, H_12-AD_-11), 1.42 (s, 3 H, H-14), 1.36 (s, 3 H, H-13), 1.35–1.30 (m, 22 H, H_12-AD_-4 to H_12-AD_-10, H_oct_-4 to H_oct_-7), 0.92 (m, 3 H, H_oct_-8); ^13^C NMR (600 MHz, methanol-*d*_4_) δ ppm = 178.71 (1 C, C-12), 174.89 (1 C, C_oct_-1), 174.83 (1 C, C_asp_-1), 174.35 (1 C, C_12-AD_-1), 173.64 (1 C, C_Asp_-4), 172.62 (1 C, C_Ac Tg_-1), 172.43 (1 C, C_Ac Asp_-1), 169.19 (1 C, C_Ang_-1), 141.65 (1 C, C-4), 140.07 (1 C, C_Ang_-3), 134.00 (1 C, C-5), 129.84 (1 C, C_Ang_-2), 86.53 (1 C, C-10), 86.33 (1 C, C-3), 80.10 (1 C, C-11), 80.05 (1 C, C-7), 79.94 (1 C, C-2), 78.62 (1 C, C-6), 68.04 (1 C, C-8), 59.66 (1 C, C-1), 51.36 (1 C, C_Asp_-2), 41.12 (1 C, C_12-AD_-12), 40.04 (1 C, C-9), 38.99 (1 C, C_Asp_-3), 36.15 (1 C, C_12-AD_-2), 35.80 (1 C, C_oct_-2), 31.27–30.70 (9 C, C_12-AD_-4 − 9 + 11, C_oct_-4 − 5), 28.60 (1 C, C_12-AD_-10), 26.58 (1 C, C_12-AD_-3), 26.20 (1 C, C_oct_-3), 24.28 (1 C, C_oct_-6), 24.01 (1 C, C-14), 23.32 (1 C, C_Ac Tg_-2), 23.10 (1 C, C_Ac Asp_-2), 21.38 (1 C, CH_3_ from C_Ang_-2), 16.69 (1 C, C_Ang_-4), 16.46 (1 C, C-13), 15.05 (1 C, C_oct_-8), 13.59 (1 C, C-15); HR-MS-ESI: [C_48_H_74_N_2_O_16_Na]^+^ *m*/*z*: 957.4930. Found 957.4931.

## 4. Conclusions

Synthetic protocols for obtaining the three thapsigargin prodrugs G114 (**2**), G115 (**4**) and Mipsagargin (**6**) were developed. In particular, the continuous manual SPPS procedure for **6** proved simple to perform compared to solution-phase procedures involving more complex protecting schemes.

## Supplementary Materials

The following are available online, Figure S1: Compound **2** (G114, TZ 83): analytical HPLC; Figure S2: Compound **2** (G114, TZ 83): ^1^H-NMR in methanol-*d*_4_; Figure S3: Compound 2 (G114, TZ 83): 13C-NMR in methanol-*d*_4_; Figure S4: Compound 2 (G114, TZ 83): COSY in methanol-*d*_4_; Figure S5: Compound 2 (G114, TZ 83): HSQC in methanol_4_; Figure S6: Compound 2 (G114, TZ 83): HMBC in methanol-*d*_4_; Figure S7: Compound 2 (G114, TZ 83): HR-MALDI; Figure S8: Compound 4 (G115, TZ 82): analyt. HPLC; Figure S9: Compound 4 (G115, TZ): 1H-NMR in methanol-*d*_4_; Figure S10: Compound 4 (G115, TZ 82): ^13^C-NMR in methanol-*d*_4_; Figure S11: Compound 4 (G115, TZ 82): COSY in methanol-*d*_4_; Figure S12: Compound 4 (G115, TZ 82): HSQC in methanol-*d*_4_; Figure S13: Compound 4 (G115, TZ 82): HMBC in methanol-*d*_4_; Figure S15: Compound 6 (TZ 70): analyt. HPLC; Figure S16: Compound 6 (TZ 70): ^1^H-NMR in methanol-*d*_4_; Figure S17: Compound 6 (TZ 70): ^13^C-NMR in methanol-*d*_4_; Figure S18: Compound **6** (TZ 70): COSY in methanol-*d*_4_; Figure S19: Compound 6 (TZ 70): HSQC in methanol-*d*_4_; Figure S20: Compound 6 (TZ 70): HMBC in methanol-*d*_4_; Figure S21: Compound 8 (TZ 81): analyt. HPLC; Figure S24: Compound 8 (TZ 81): COSY NMR in methanol-*d*_4_; Figure S25: Compound 8 (TZ 81): HSQC in methanol-*d*_4_; Figure S26: Compound 8 (TZ 81): HR-MS.
