# Supplementary material for: Preparation of Enzyme-Activated Thapsigargin Prodrugs by Solid-Phase Synthesis
Source: Molecules. 2018 Jun 15;23(6):1463. doi: 10.3390/molecules23061463 (PMC6100299; doi:10.3390/molecules23061463)
Supplement: Supplementary file 1 [file molecules-23-01463-s001.pdf]

Supplementary information for

Preparation of Tumor-Targeted Thapsigargin Prodrugs by Solid-Phase Synthesis

Tomas Zimmermann [1,4], Søren Brøgger Christensen [2] and Henrik Franzyk [3,*]

[1] Department of Drug Design and Pharmacology, University of Copenhagen; tomas.zimmermann@vscht.cz
[2] Department of Drug Design and Pharmacology, University of Copenhagen; soren.christensen@sund.ku.dk
[3] Department of Drug Design and Pharmacology, University of Copenhagen; henrik.franzyk@sund.ku.dk
[4] Present address: Jagtvej 162, København Ø, DK-2100
* henrik.franzyk@sund.ku.dk; Tel.: +45-3533-6255

Contents:

Compound **2** (G114, TZ 83)	Analyt. HPLC	S2
Compound **2** (G 114, TZ 83)	NMR spectra	S3 - S7
Compound **2** (G 114, TZ 83)	HRMS	S8
Compound **4** (G 115, TZ 82)	Analyt. HPLC	S9
Compound **4** (G 115, TZ 82)	NMR spectra	S10 - S14
Compound **4** (G 115, TZ 82)	HRMS	S15
Compound **6** (G202 TZ70)	Analyt. HPLC	S16
Compound **6** (G202 TZ70)	NMR spectra	S17 - S21
Compound **8** (TZ 81)	Analyt. HPLC	S22
Compound **8** (TZ 81)	NMR spectra	S23 - S26
Compound **8** (TZ 81)	HRMS	S27

Compound **2** (G114,TZ 83)

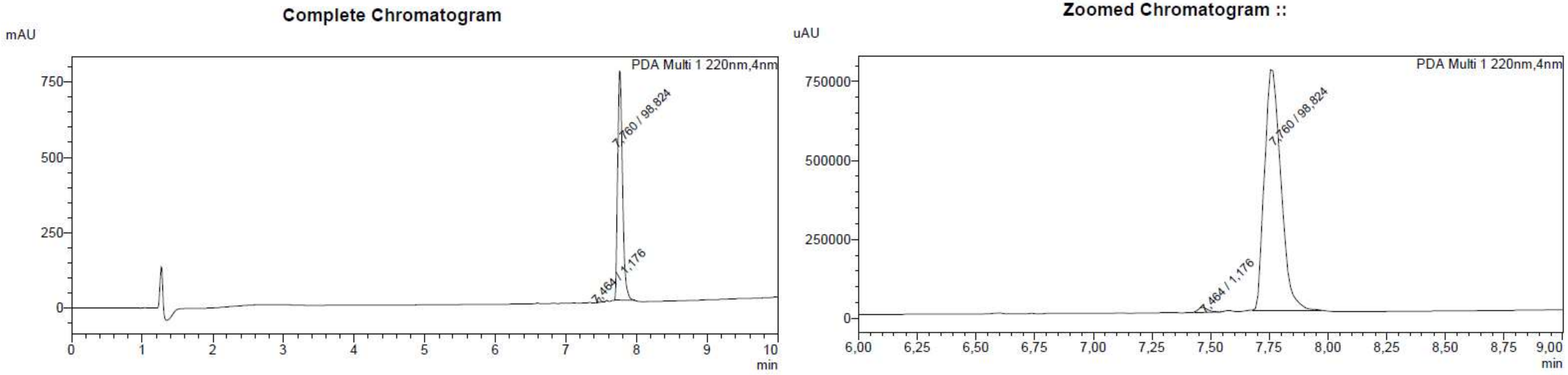

PDA Ch1 220nm

Peak#	Ret. Time	Area%	Height	Area
1	7,464	1,176	16253	44207
2	7,760	98,824	760065	3714051
Total		100,000	776318	3758258

Figure S1: Compound **2** (G114, TZ 83): analyt. HPLC

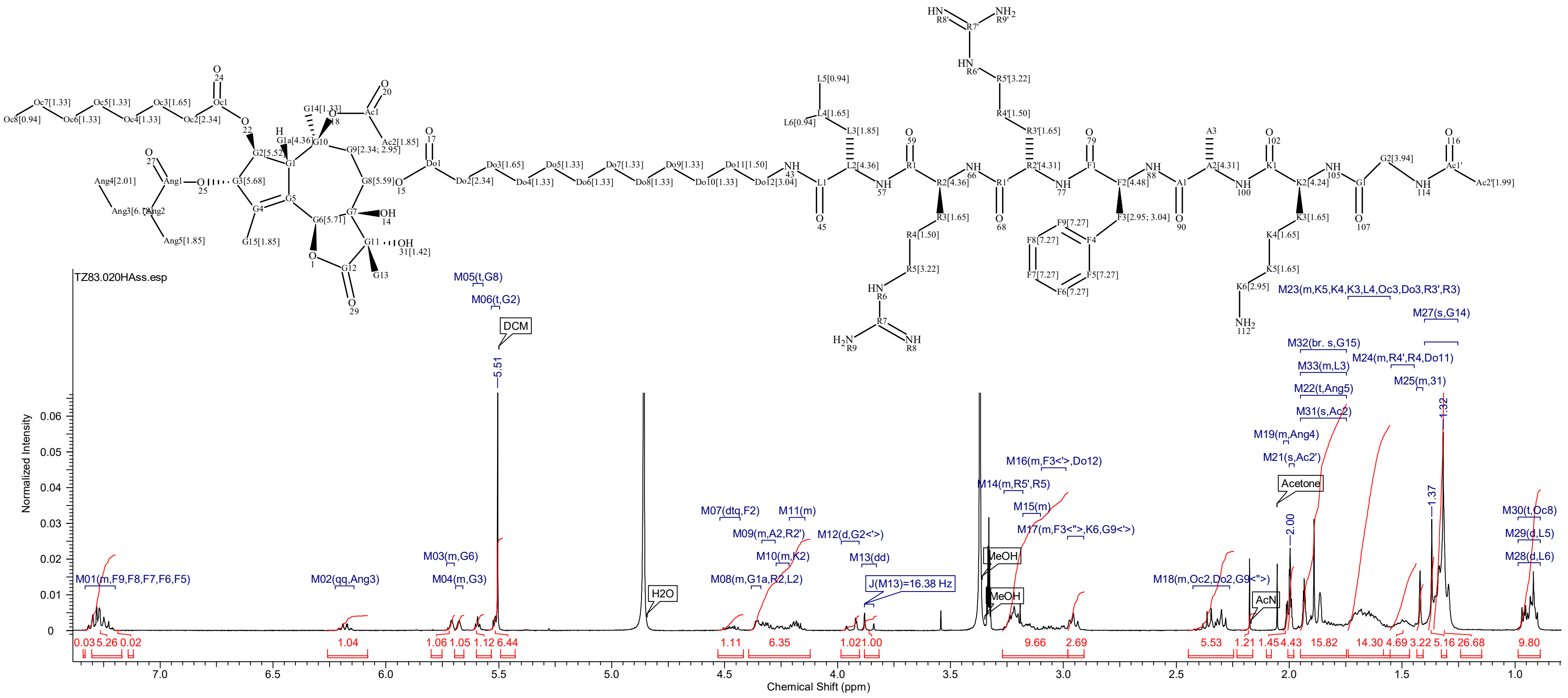

Figure S2: Compound **2** (G114, TZ 83): ^{1}H NMR in methanol-d$_4$

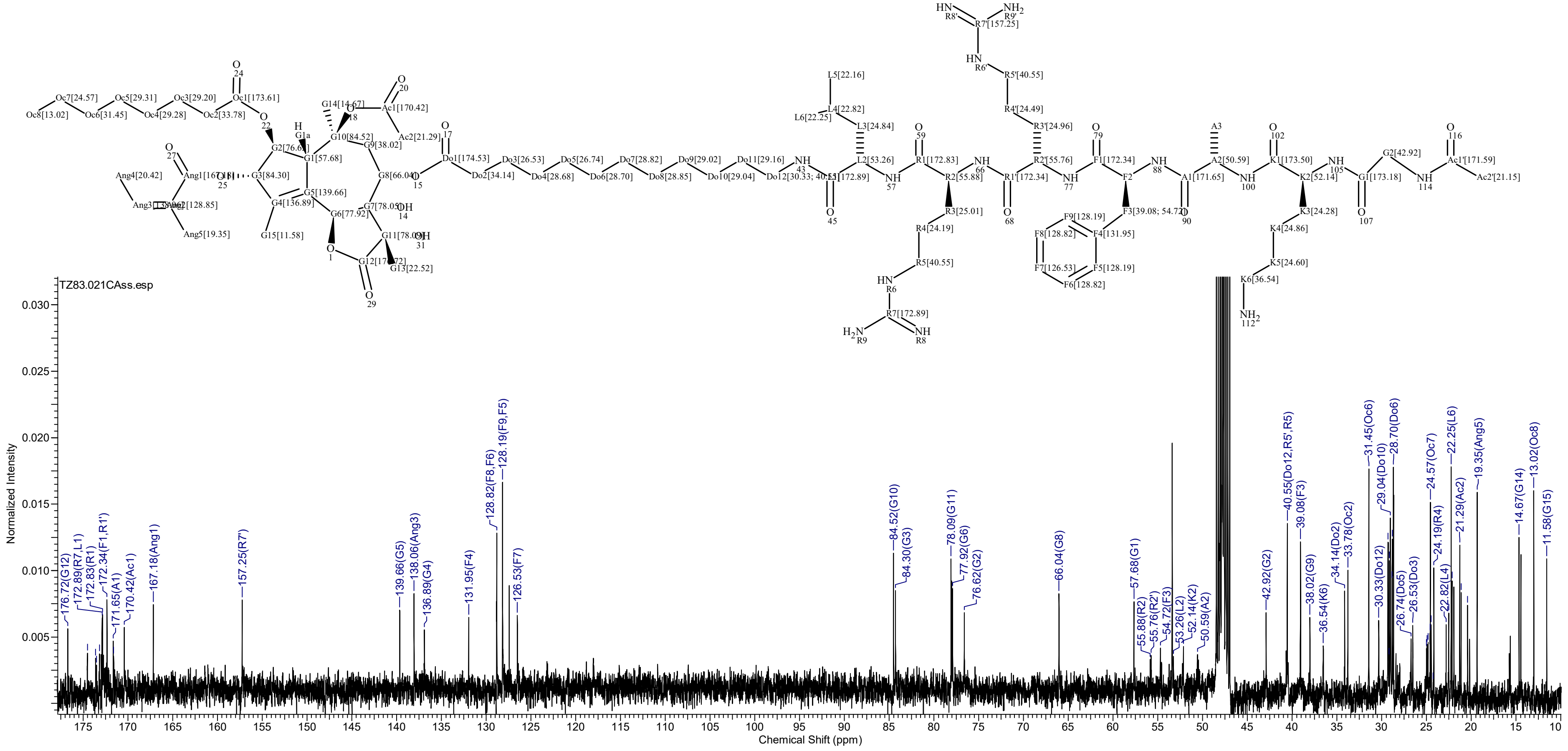

Figure S3: Compound **2** (G114, TZ 83): ^{13}C NMR in methanol-d$_4$

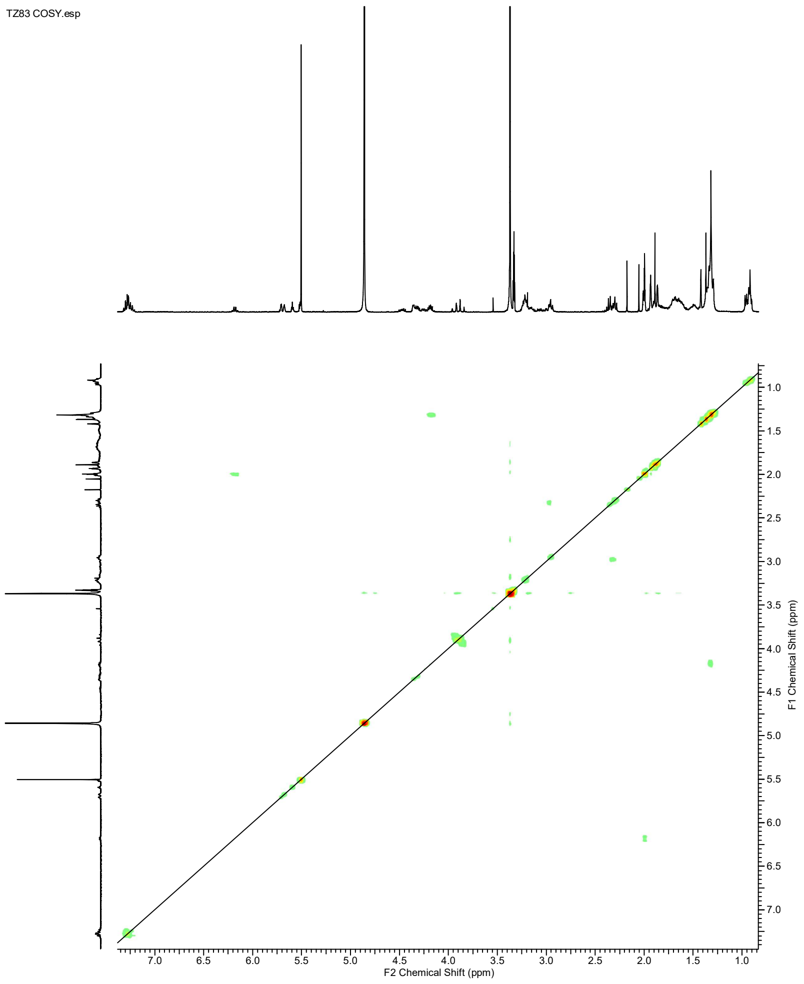

Figure S4: Compound **2** (G114, TZ 83): COSY in methanol-d_4

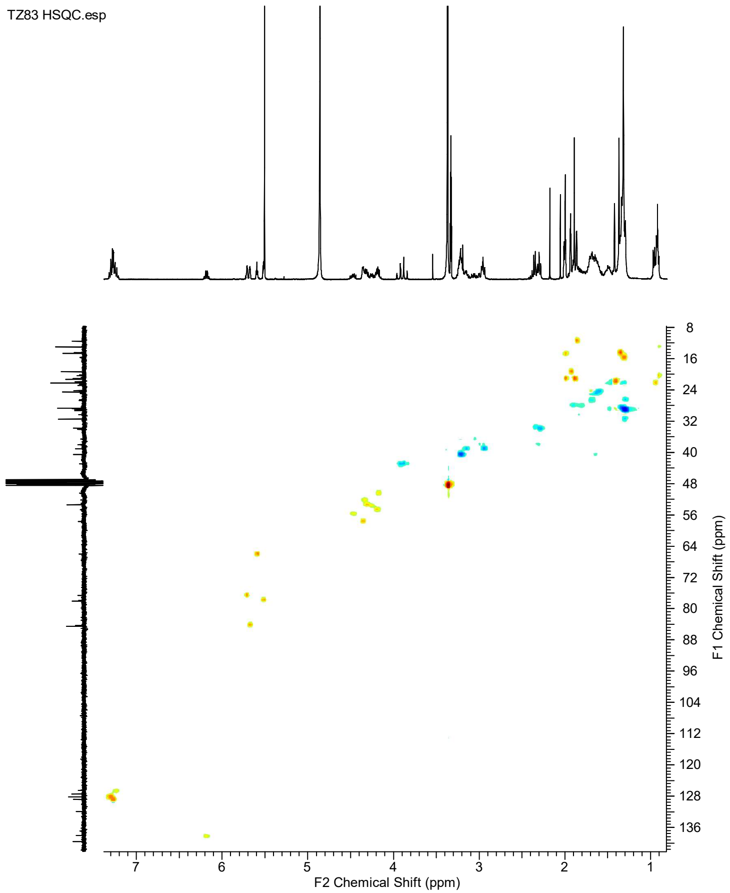

Figure S5: Compound **2** (G114, TZ 83): HSQC in methanol-d_4

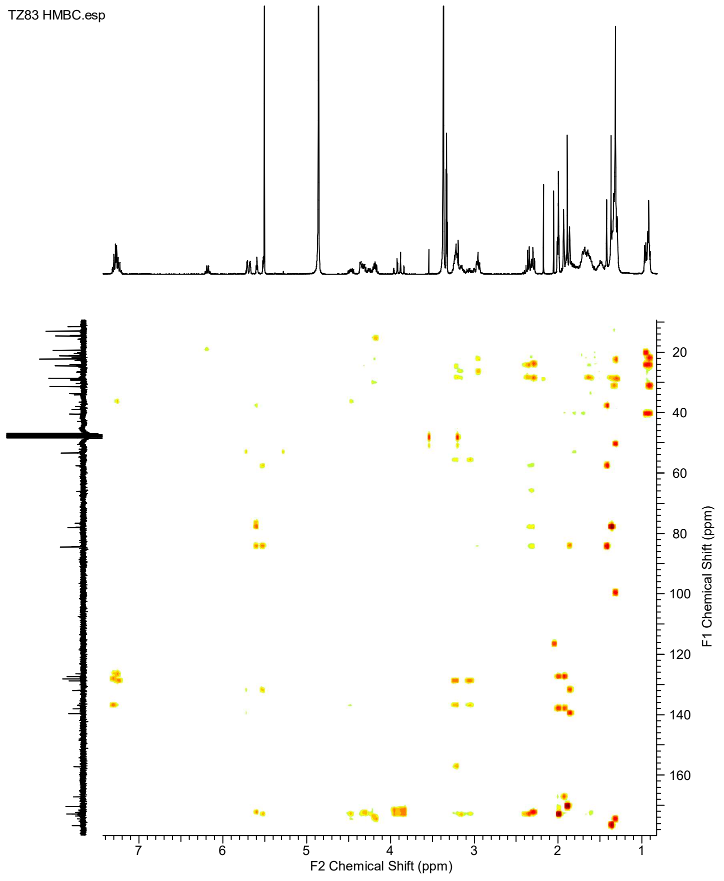

Figure S6: Compound **2** (G114, TZ 83): HMBC in methanol-d_4

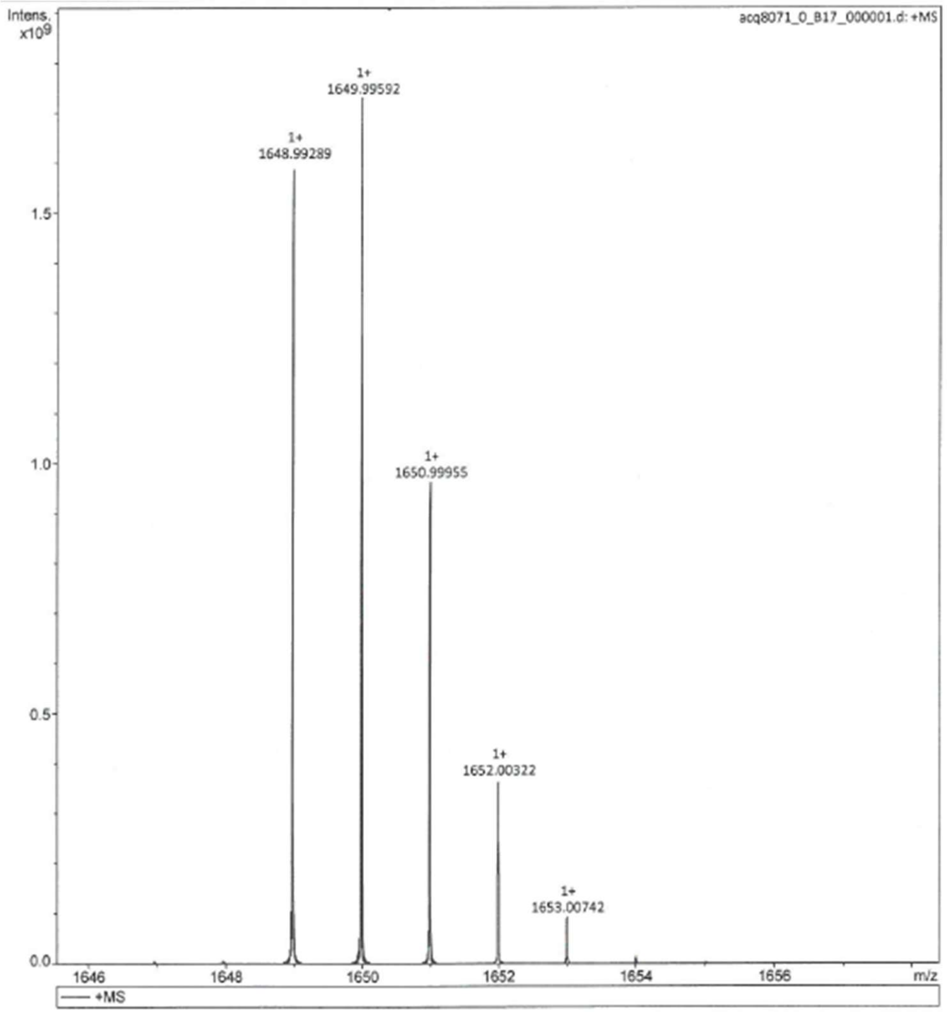

Figure S7: Compound **2** (G114, TZ 83): HR-MALDI

Compound **4** (G115, TZ 82)

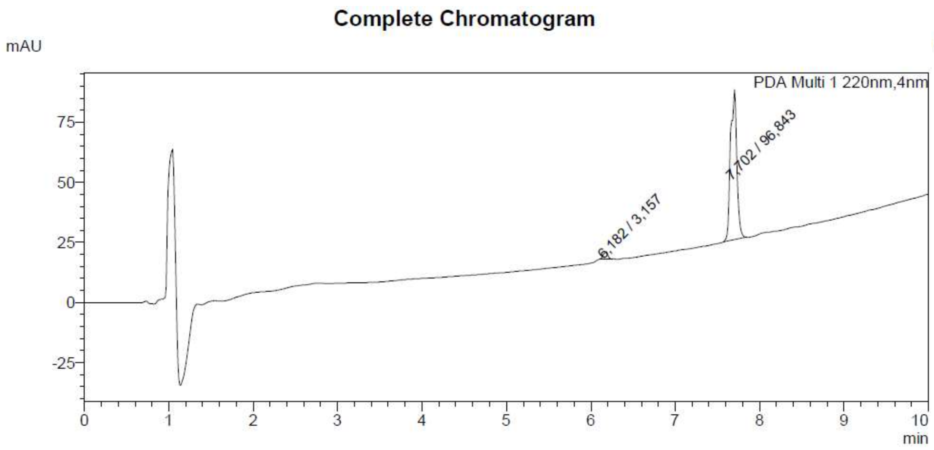

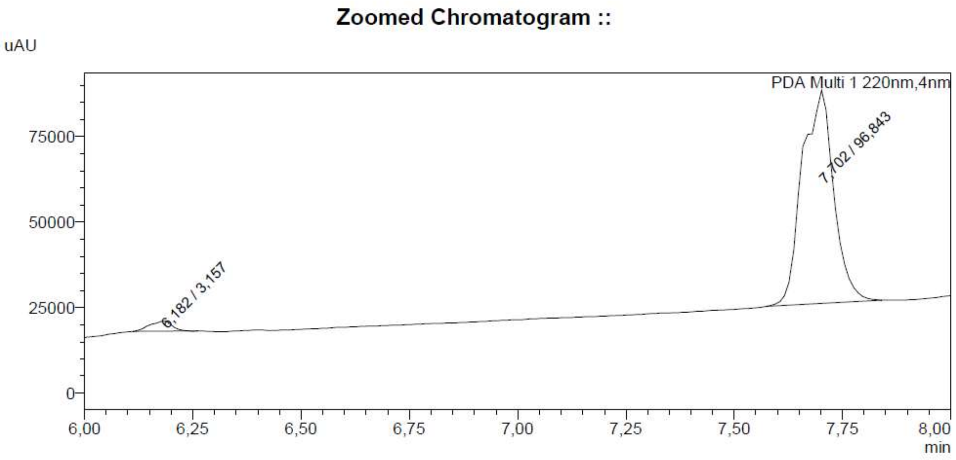

PDA Ch1 220nm

Peak#	Ret. Time	Area%	Height	Area
1	6,182	3,157	2945	10337
2	7,702	96,843	62310	317105
Total		100,000	65255	327443

Figure S8: Compound **4** (G115, TZ 82): analyt. HPLC

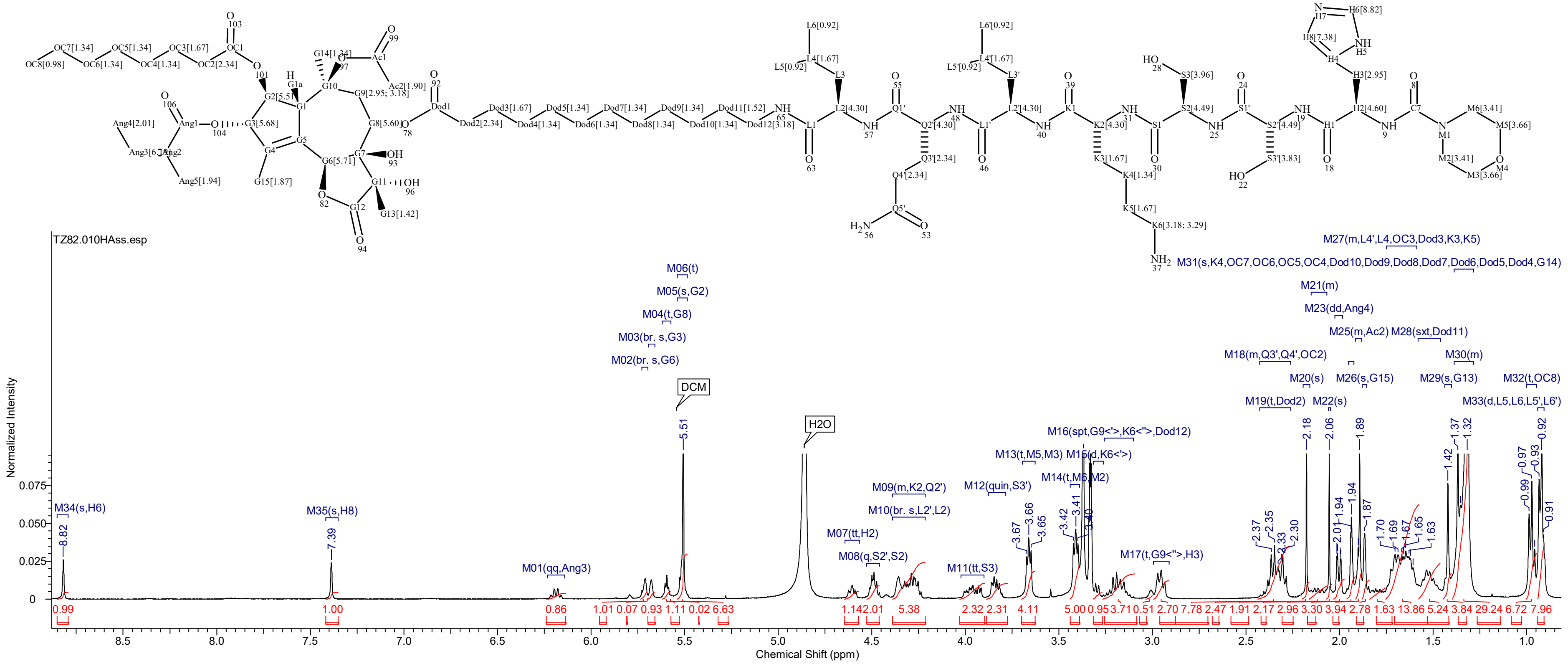

Figure S9: Compound **4** (G115, TZ): ^{1}H NMR in methanol-d_4

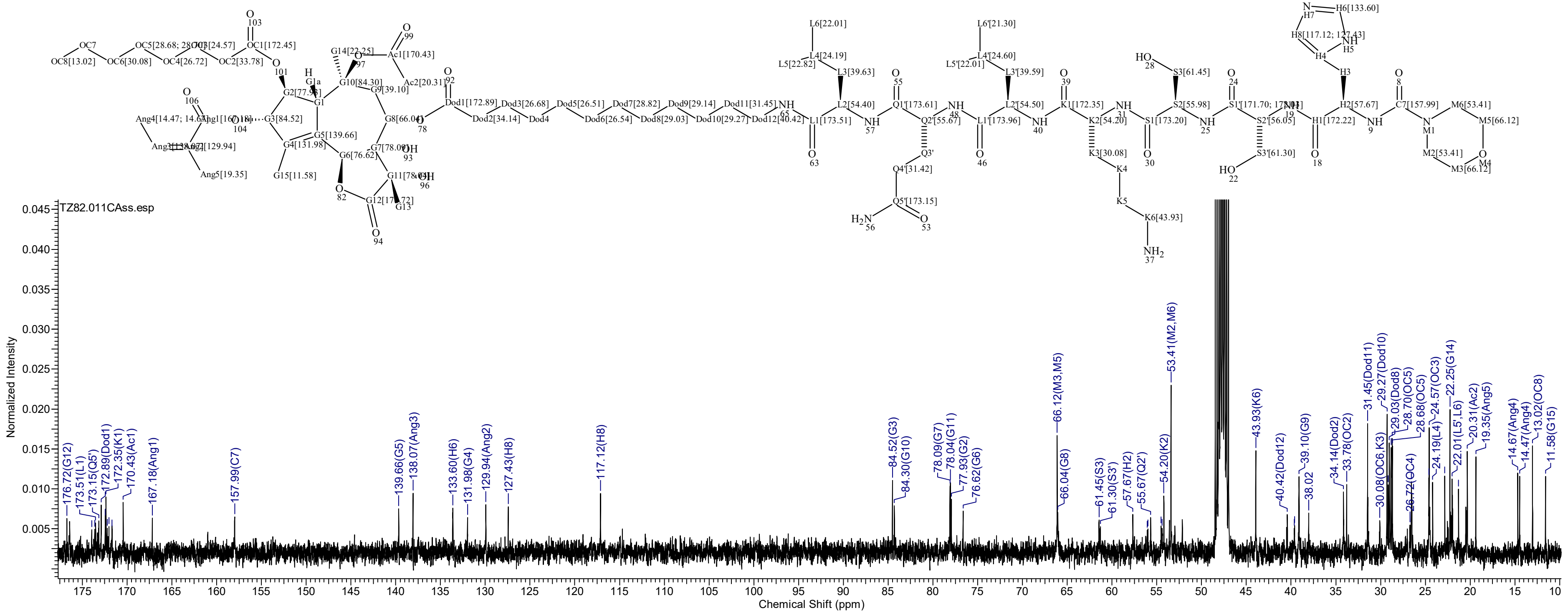

Figure S10: Compound **4** (G115, TZ 82): ^{13}C NMR in methanol-d_4

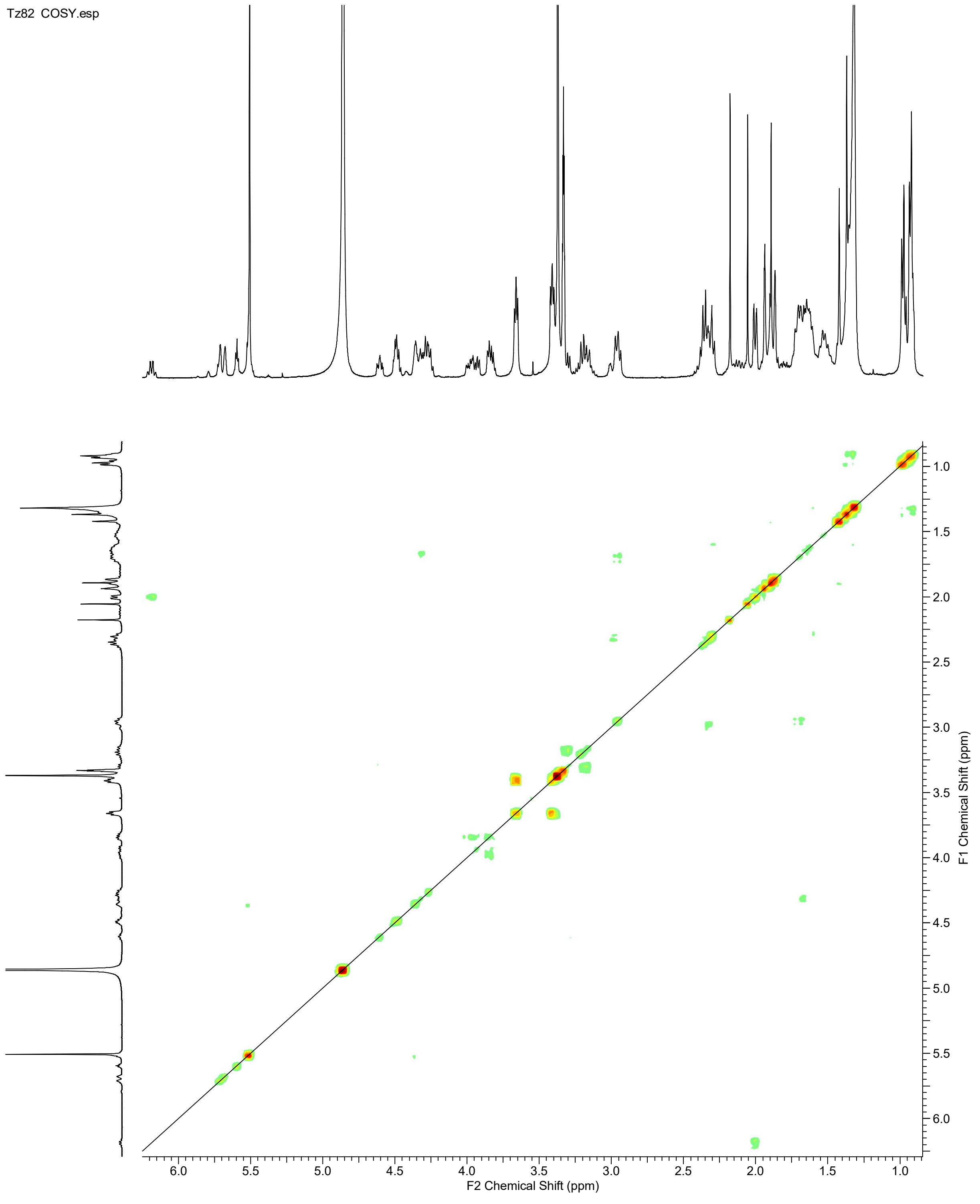

Figure S11: Compound **4** (G115, TZ 82): COSY in methanol-d_4

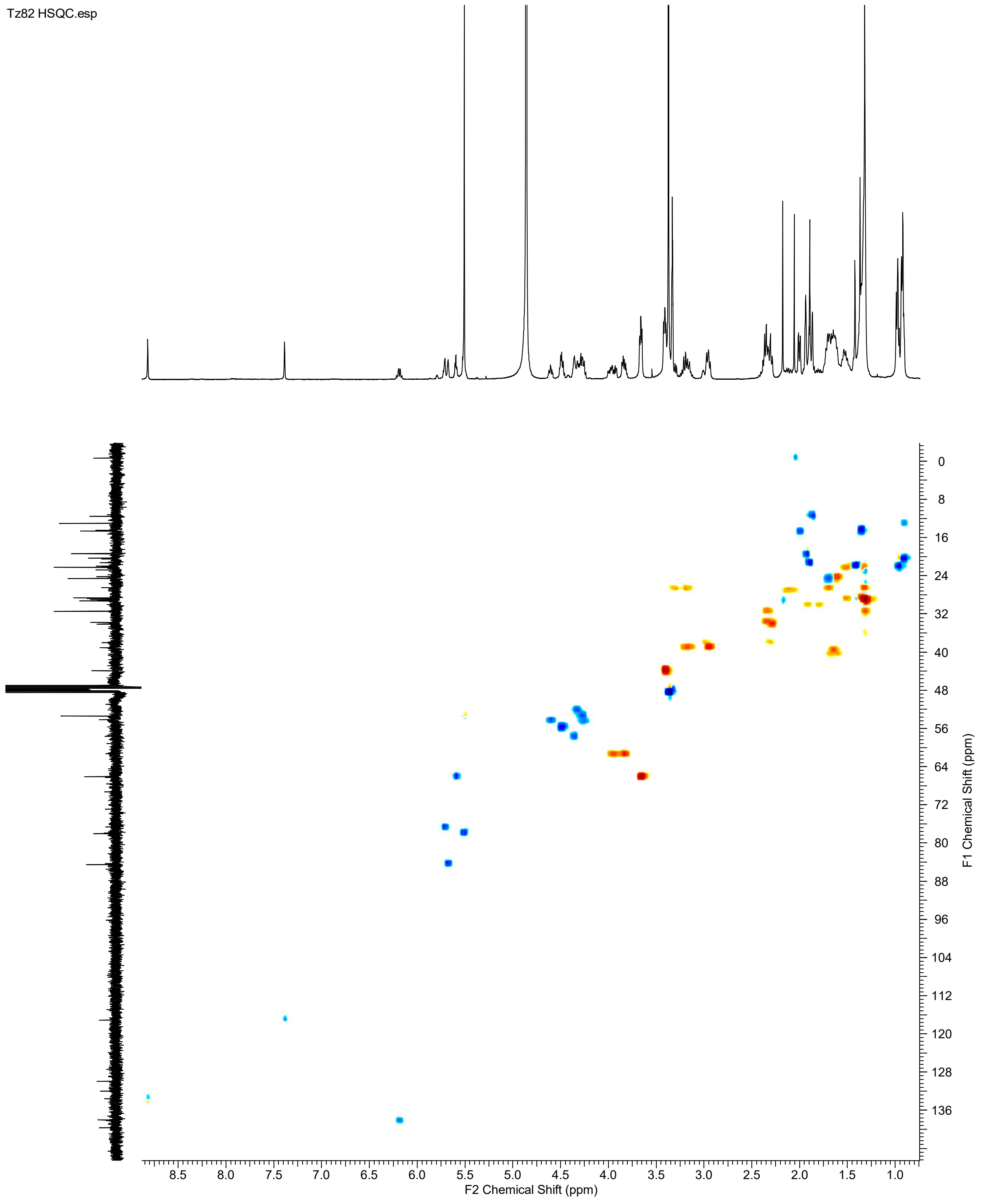

Figure S12: Compound **4** (G115, TZ 82): HSQC in methanol-d_4

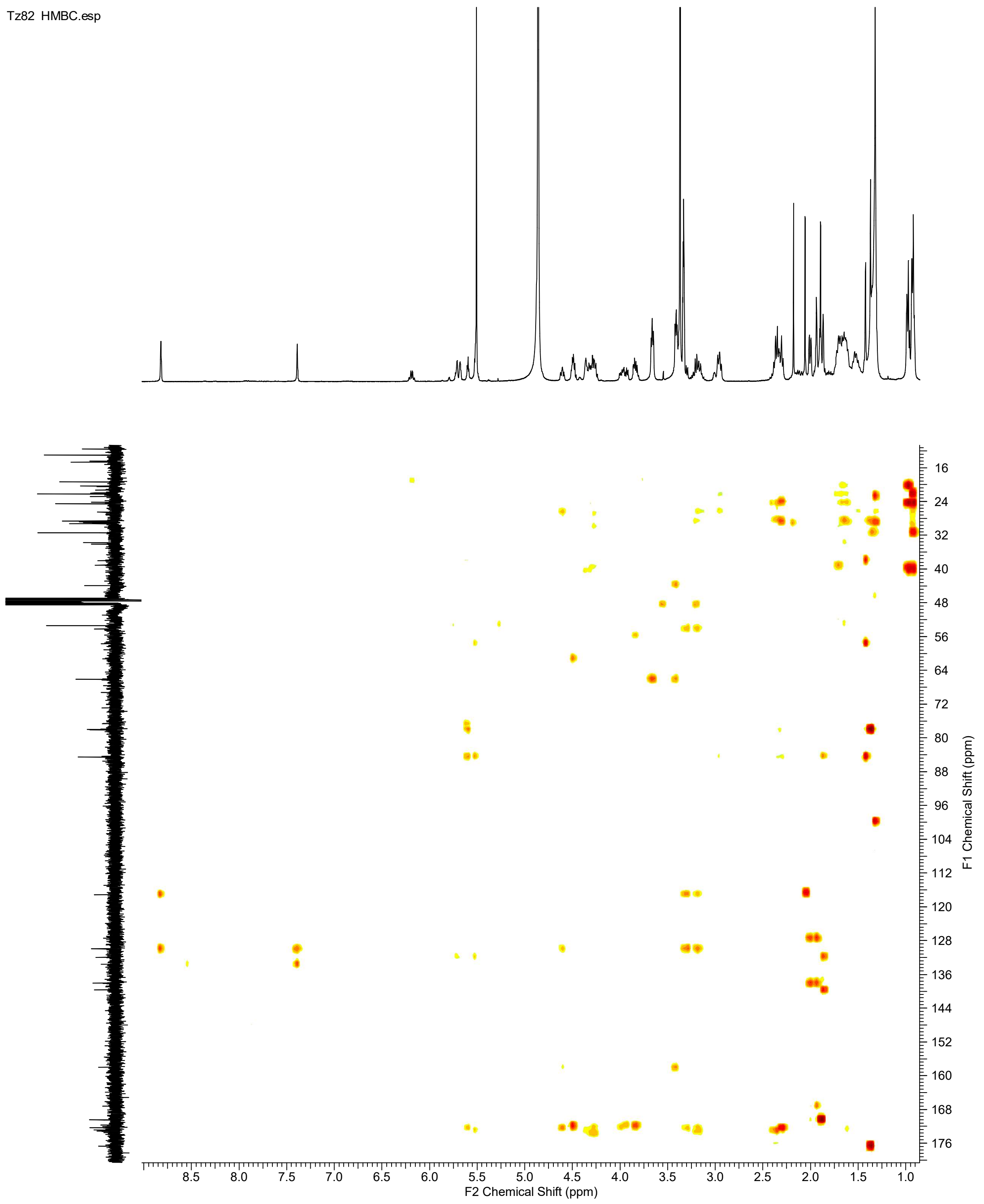

Figure S13: Compound **4** (G115, TZ 82): HMBC in methanol-d_4

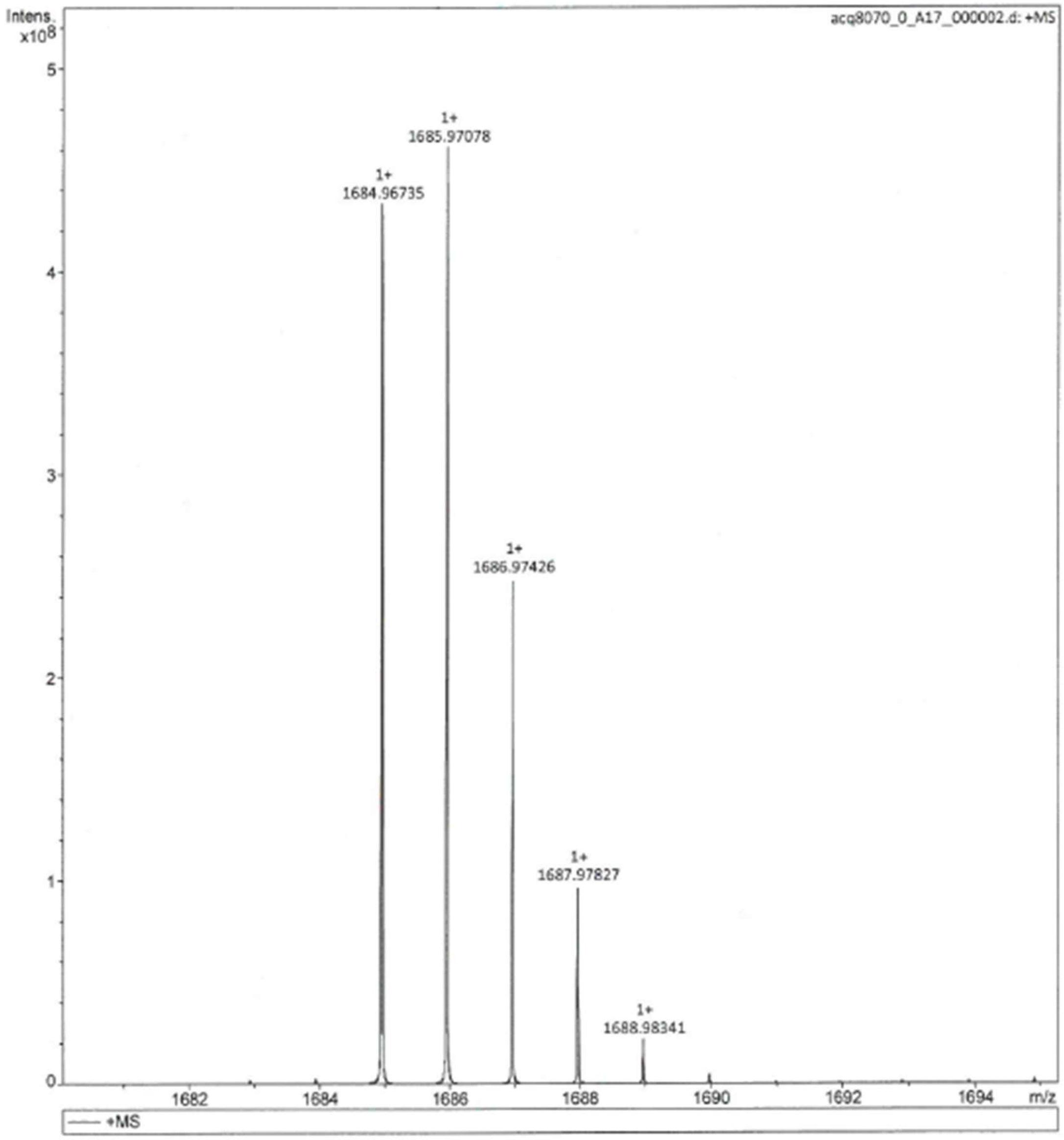

Figure S14: Compound **4** (G115, TZ 82): HR-MALDI

Compound **6** (G202, TZ70)

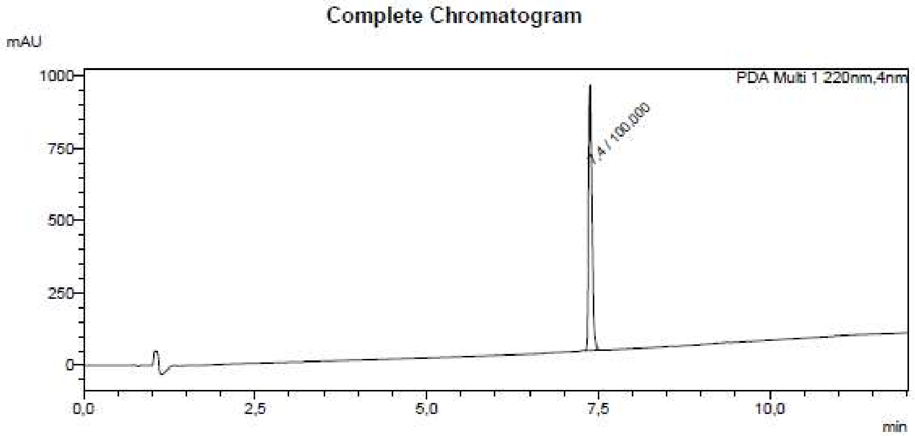

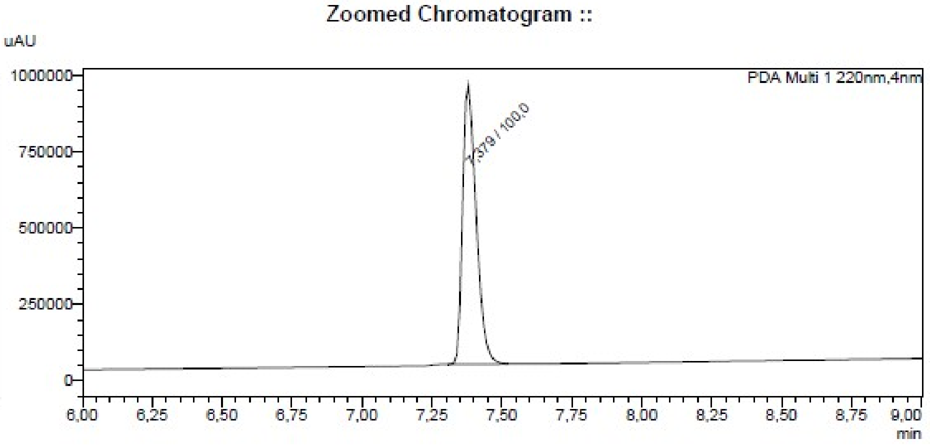

PDA Ch1 220nm

Peak#	Ret. Time	Area%	Height	Area
1	7,379	100,000	916338	3121303
Total		100,000	916338	3121303

Figure S15: Compound **6** (TZ 70): analyt. HPLC

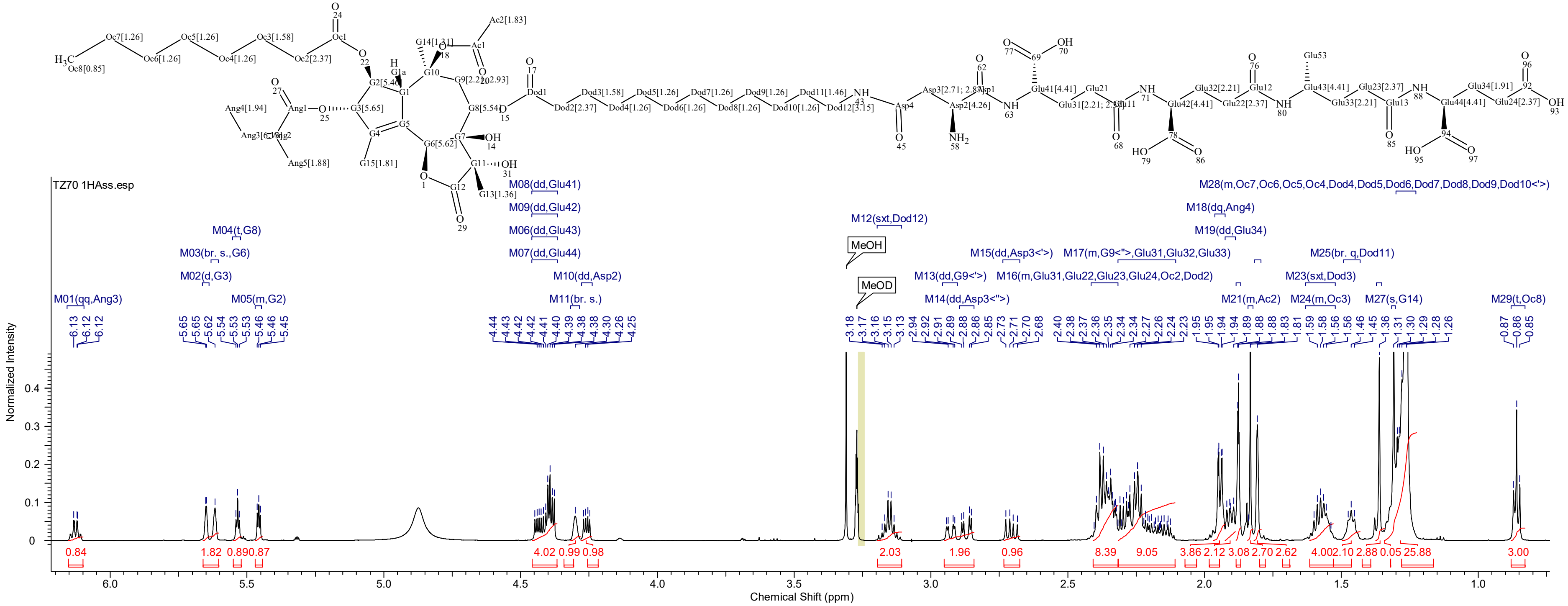

Figure S16: Compound **6** (TZ 70): ^{1}H NMR in methanol-d4

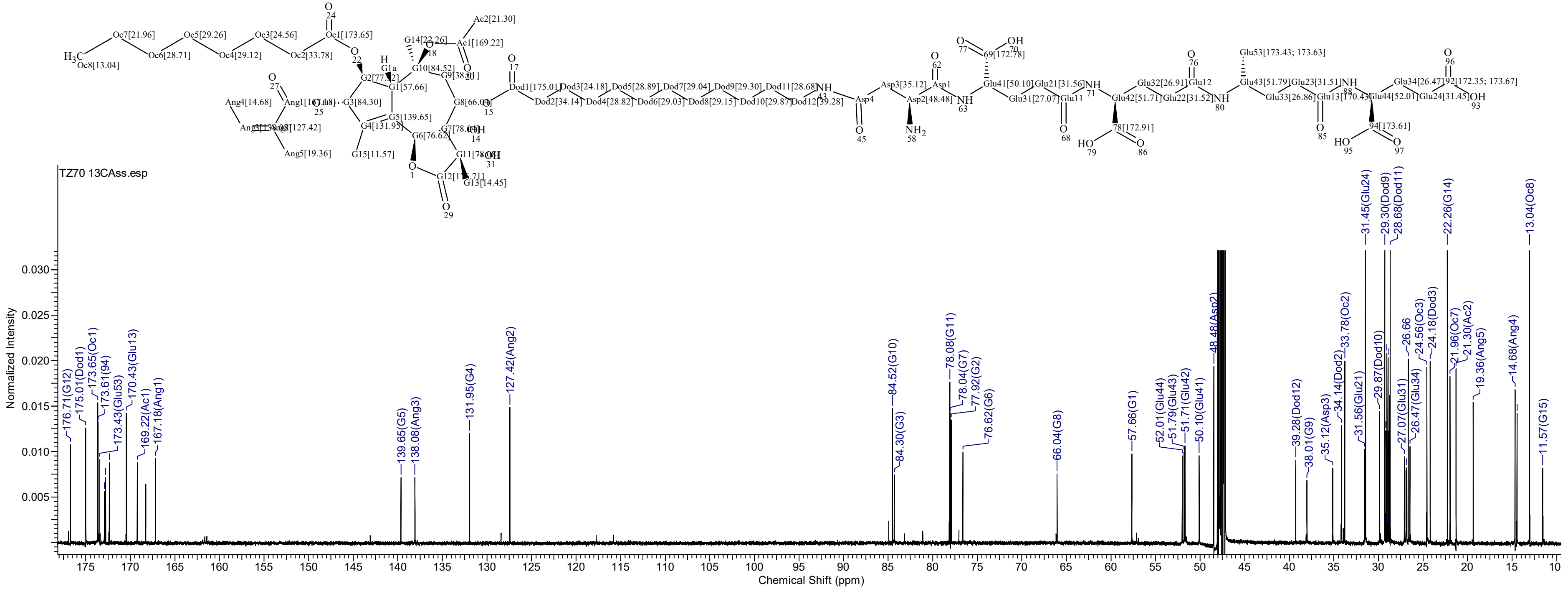

Figure S17: Compound **6** (TZ 70): ^{13}C NMR in methanol-d4

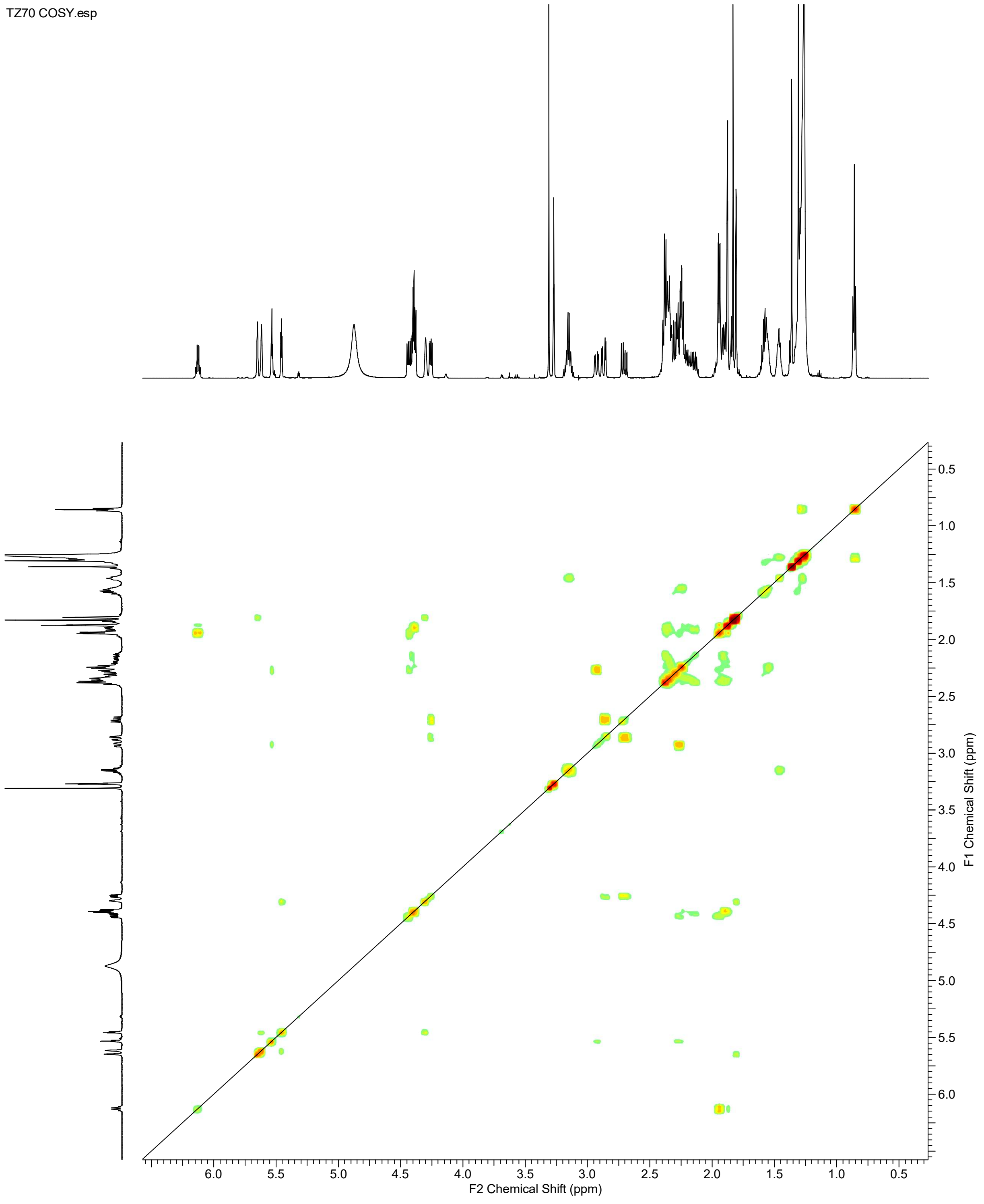

Figure S18: Compound **6** (TZ 70): COSY in methanol-d_4

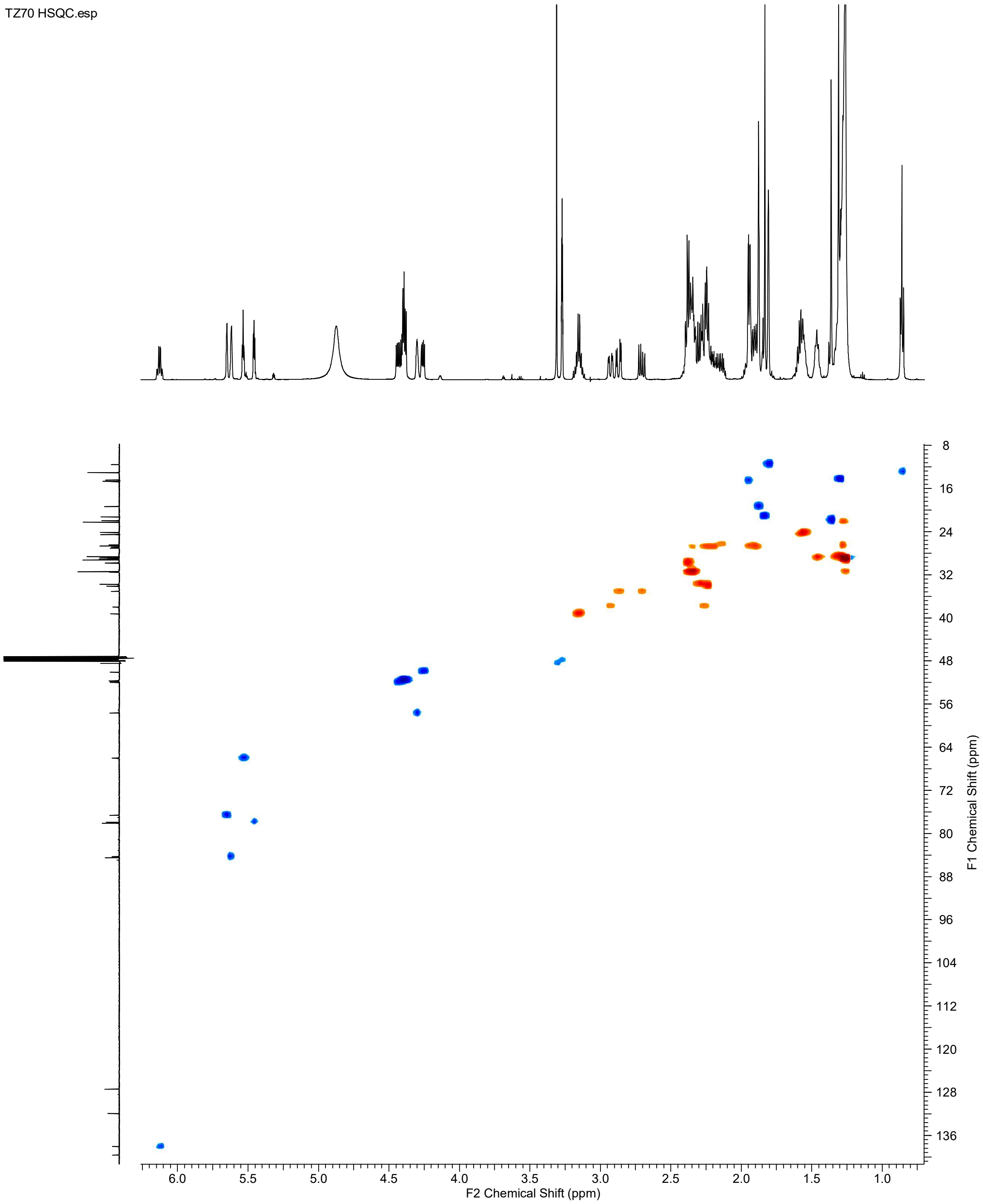

Figure S19: Compound **6** (TZ 70): HSQC in methanol-d_4

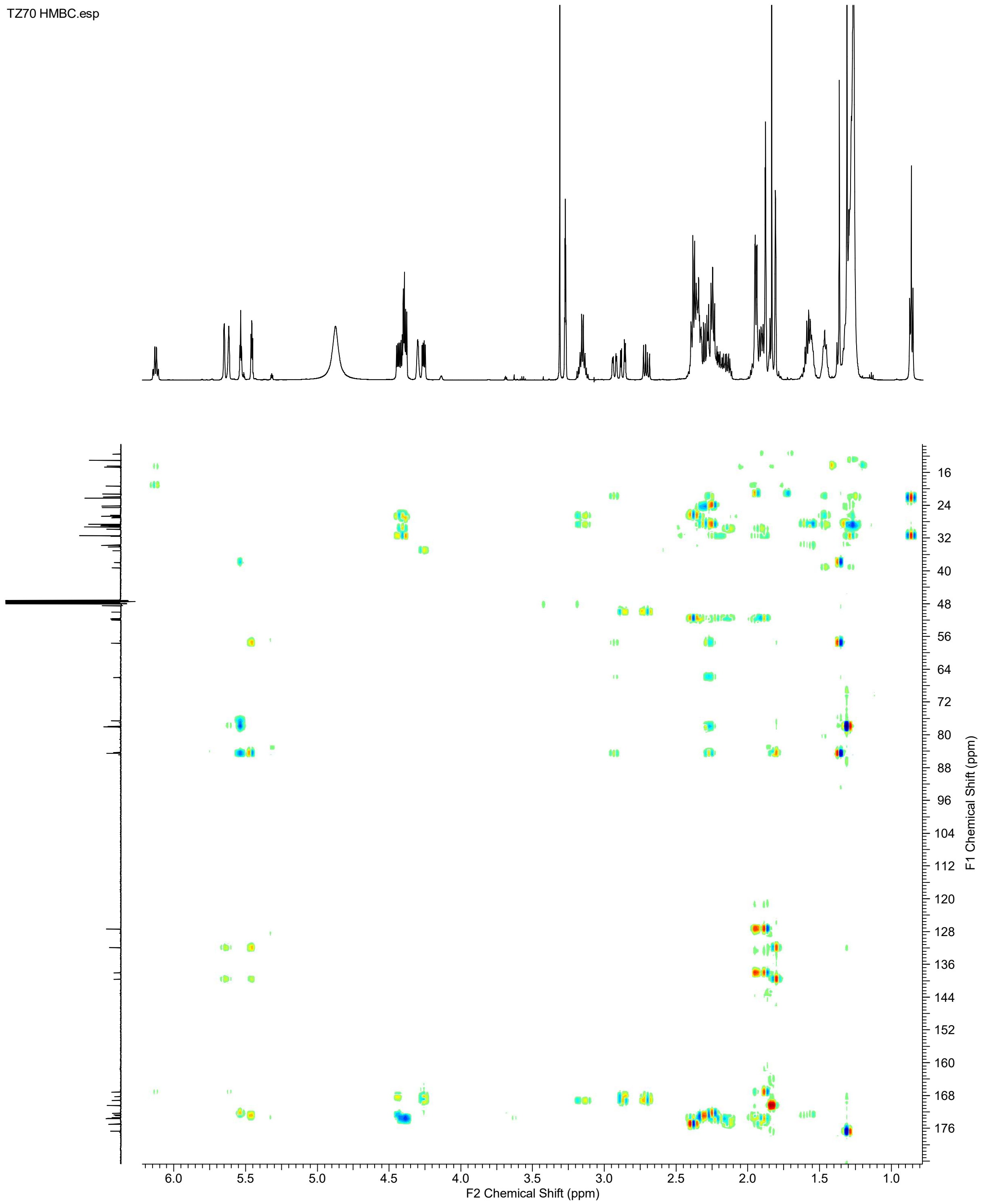

Figure S20: Compound **6** (TZ 70): HMBC in methanol-d_4

Compound **8** (TZ 81)

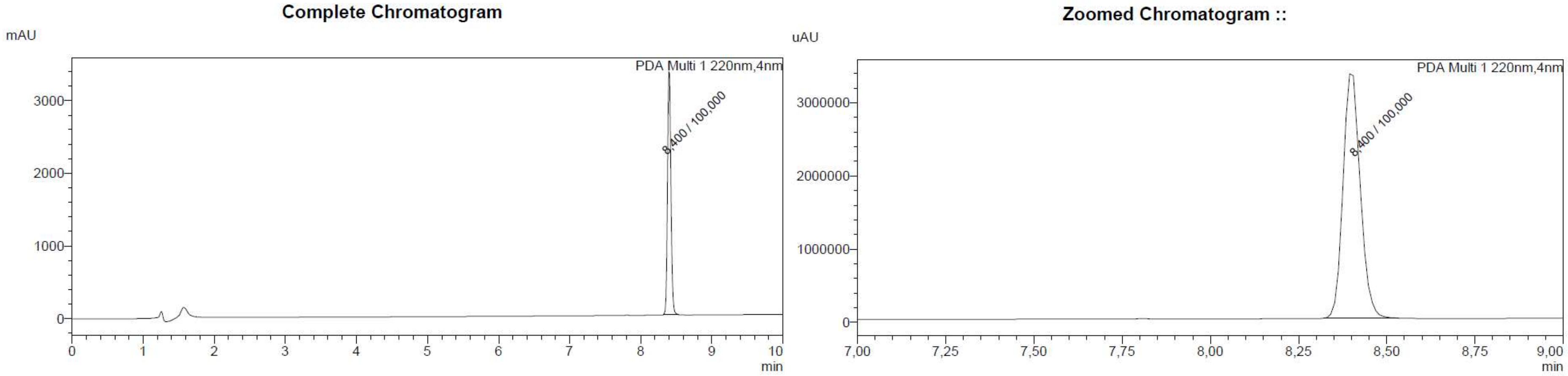

PDA Ch1 220nm

Peak#	Ret. Time	Area%	Height	Area
1	8,400	100,000	3341564	11446141
Total		100,000	3341564	11446141

Figure S21: Compound **8** (TZ 81): analyt. HPLC

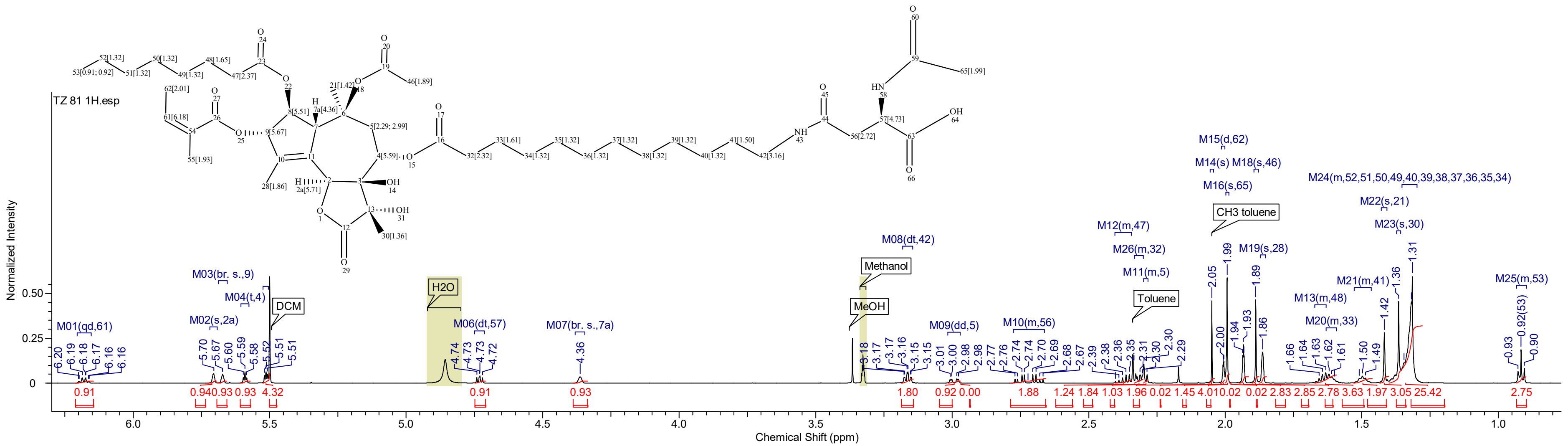

Figure S22: Compound **8** (TZ 81): ^{1}H NMR in methanol-d$_4$

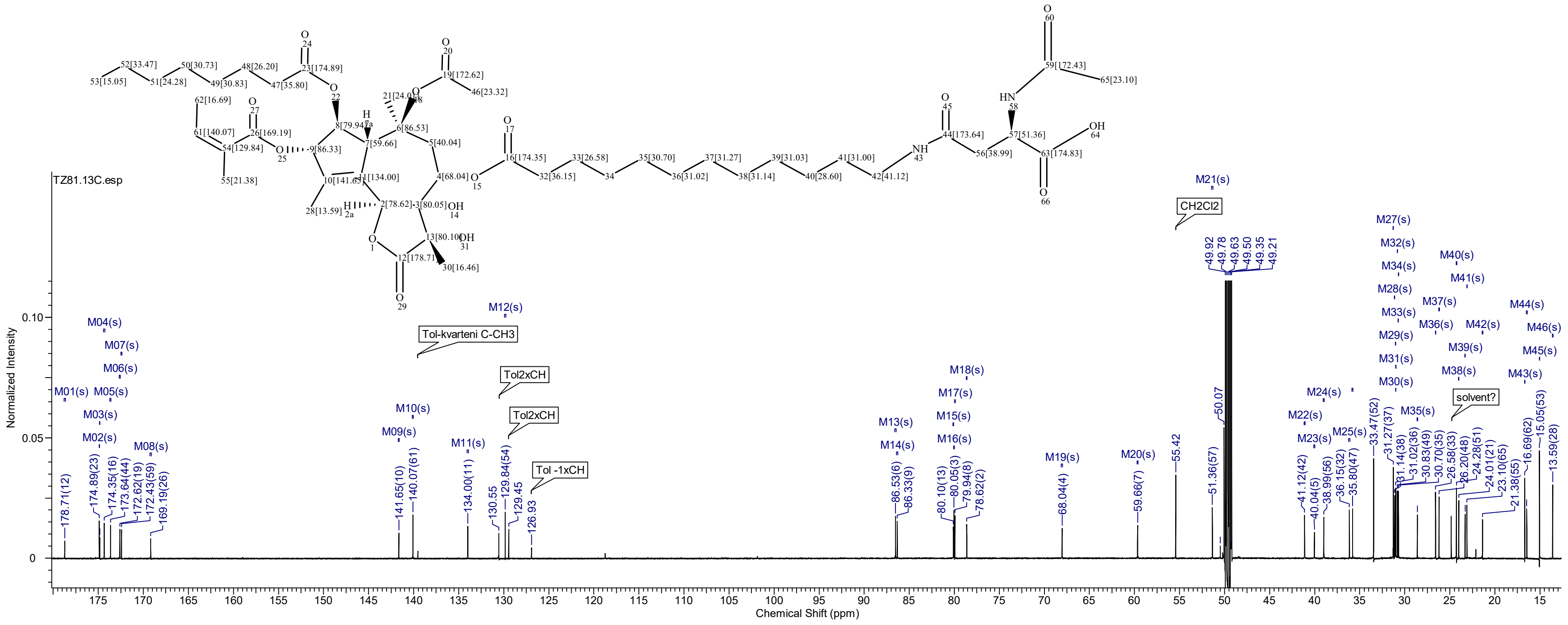

Figure S23: Compound **8** (TZ 81): ^{13}C NMR in methanol-d$_4$

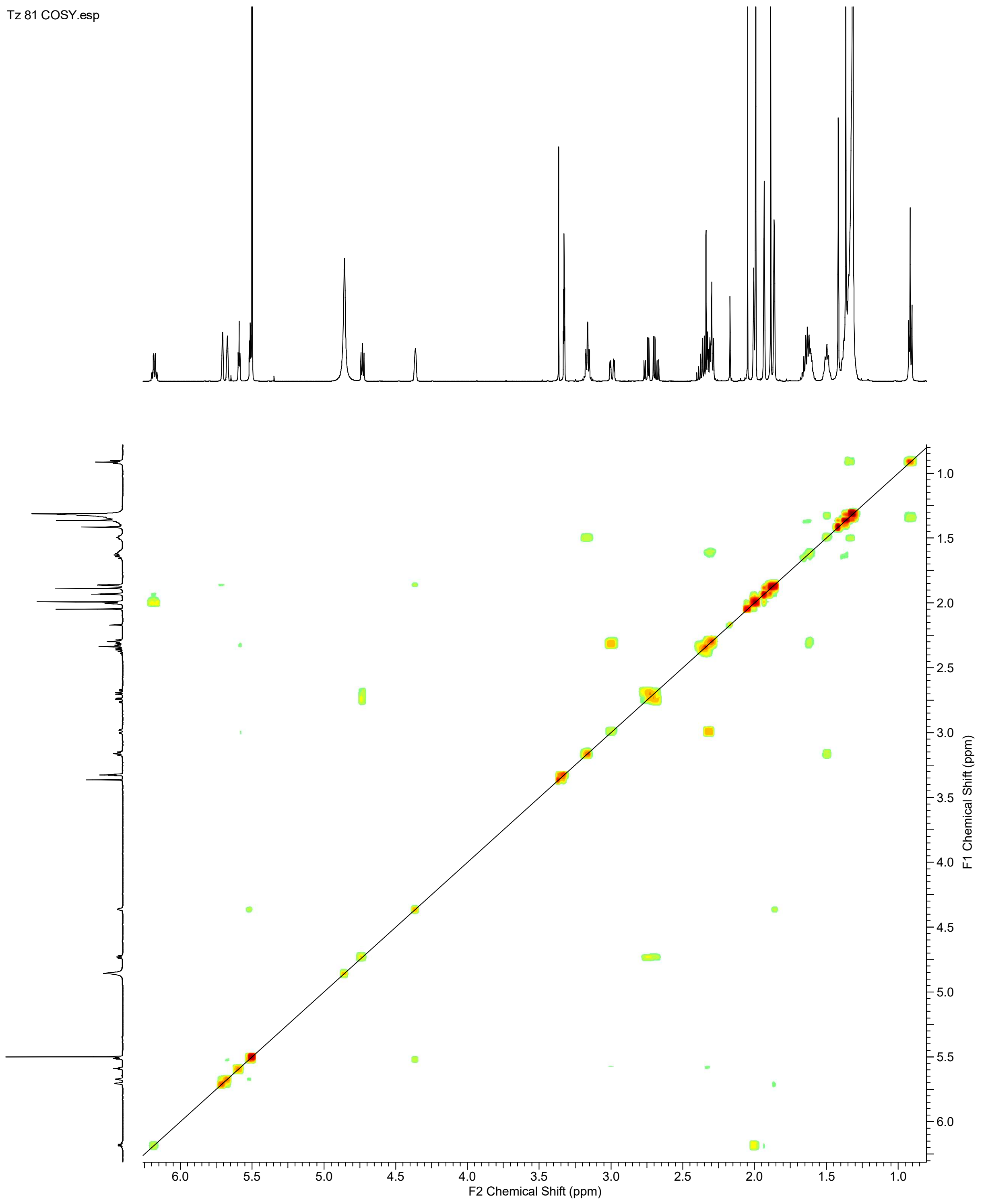

Figure S24: Compound **8** (TZ 81): COSY NMR in methanol-d_4

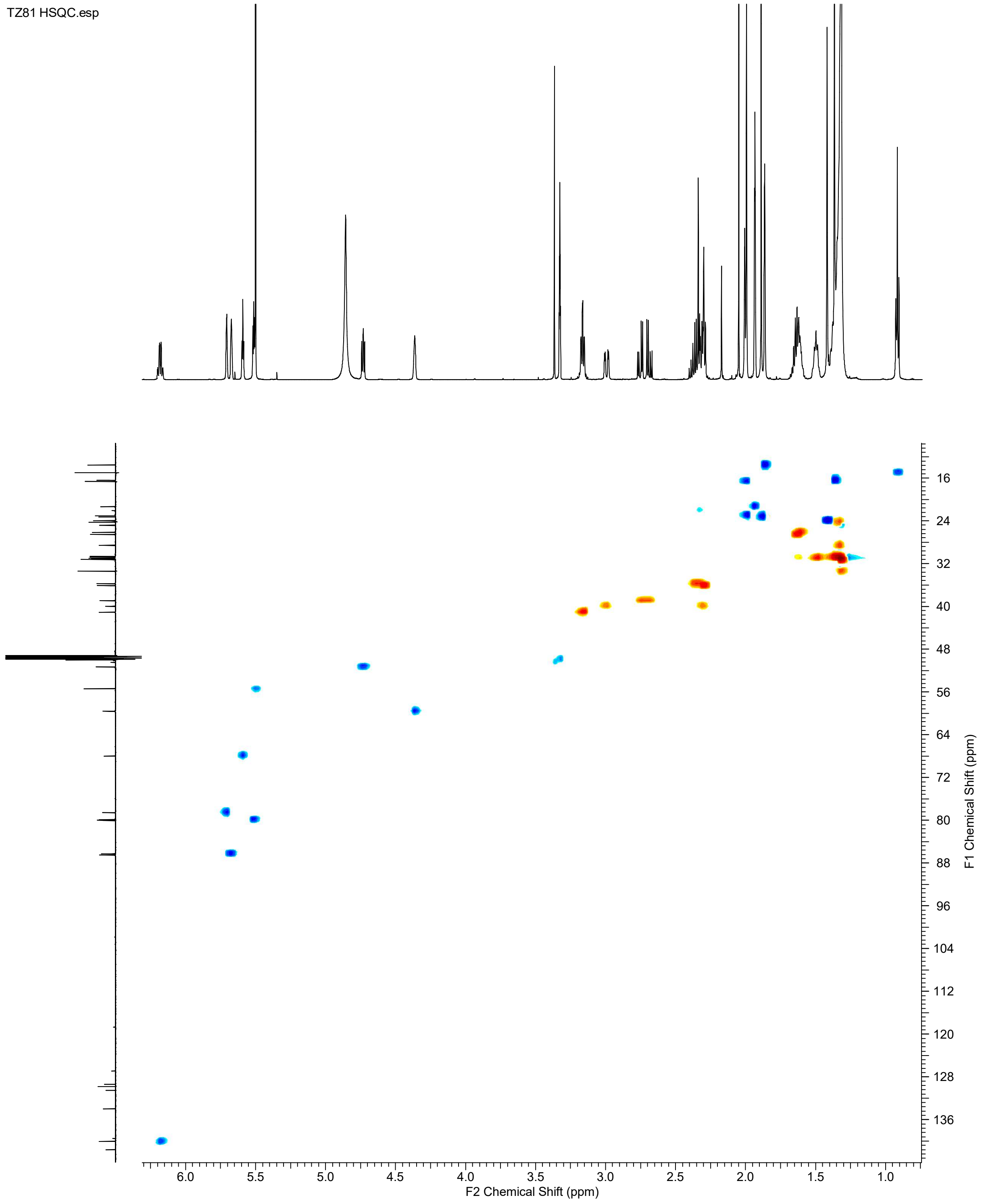

Figure S25: Compound **8** (TZ 81): HSQC NMR in methanol-d_4

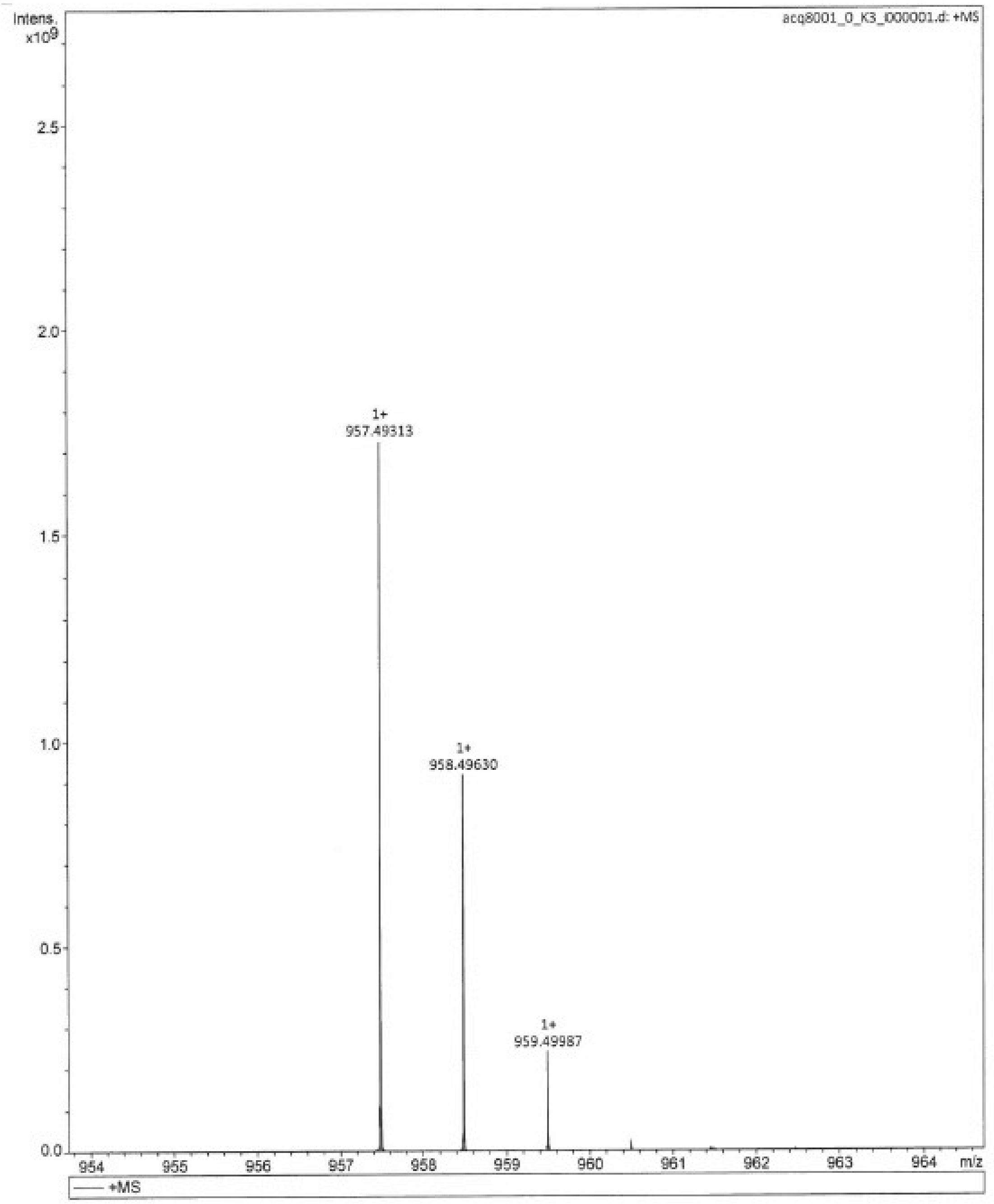

Figure S26: Compound **8** (TZ 81): HR-MS
